# What influences preparations of discharge documentation at patient discharge? An interview study with hospital health professionals based on the theoretical domains framework

**DOI:** 10.1136/bmjopen-2024-090753

**Published:** 2025-06-16

**Authors:** Ola Malgorzata Markiewicz, Aarya Menon, Amish Acharya, Danielle D’Lima, Fabiana Lorencatto, Ara Darzi, G Judah

**Affiliations:** 1 Department of Surgery & Cancer, Imperial College London, London, UK; 2 Centre for Health Policy, Imperial College London Institute of Global Health Innovation, London, UK; 3 Department of Applied Research London, University College London, London, UK; 4 Centre for Behaviour Change, University College London, London, UK; 5 Imperial College London, London, UK

**Keywords:** Health policy, Primary Care, Quality in health care, Hospital to Home Transition, Hospitals

## Abstract

**Abstract:**

**Objectives:**

Poor quality handover instructions prepared by hospital staff have been identified as a key threat to safe discharges. To optimise patient safety, it is important to identify and understand the influences on how discharge documentation is prepared by hospital staff. The aim of this study was to systematically identify and explore important barriers and enablers to the preparation of high-quality discharge documentation by healthcare professionals (HCPs) for primary care teams at patient discharge.

**Setting and participants:**

HCPs from different staff groups (doctors, nurses, pharmacists, occupational and physiotherapists) participated in online interviews.

**Design:**

Semistructured interviews informed by the theoretical domains framework (TDF), to identify key influences on the preparation of discharge documentation. Anonymised transcripts were analysed thematically using a combined inductive-deductive approach. Themes were framed as influences on the preparation of discharge documentation. The likely importance of influences was decided through iterative team discussions structured on predetermined criteria. Criteria included whether the influence was an existing enabler, whether beliefs about the influences were expressed voluntarily and how often they were mentioned.

**Results:**

12 HCPs were interviewed (5 junior doctors, 1 advanced nurse practitioner, 2 nurses, 1 occupational therapist, 1 physiotherapist and 2 pharmacists). Of 44 influences identified, 10 were deemed most important in the preparation of discharge documentation, spread across five TDF domains: knowledge (eg, lack of awareness of guidelines), skills (experience of hospital staff), social and professional role and identity (effective team communication), environmental context and resources (eg, software limitations) and social influences (eg, lack of feedback).

**Conclusions:**

This study identified 10 important influences on how discharge documentation is prepared by hospital staff. These influences are potential targets for subsequent interventions to improve the quality of discharge documentation and patient safety during discharge.

**STRENGTHS AND LIMITATIONS OF THIS STUDY**

- A structured approach to data collection and analysis based on a validated behavioural science framework, the theoretical domains framework (TDF), enabled a comprehensive exploration of possible influences on the preparation of discharge documentation by hospital staff for primary care teams on patient discharge.
- The heterogeneous sample of participants from different professional backgrounds provided a comprehensive overview of influences on the preparation of several disparate types of discharge documentation as part of the handover process between hospital staff and primary care teams.
- The heterogeneity of participants, however, limited the capacity to explore themes within professional groups involved in different kinds of discharge documentation, and therefore comparisons of themes between professional groups were not possible.
- The use of the TDF to structure the interview guide is likely to have led to more direct questioning of participants, which may have consequently contributed to influences being mentioned by participants that would not have been otherwise, potentially inflating their importance.

## Introduction

 With the UK National Health Service (NHS) experiencing unprecedented demand for beds, there is pressure for staff to discharge patients from hospital sooner.^1^,^3^ Care in the NHS can be delivered in hospitals, and inpatient care involves an admission to a hospital bed. Following discharge from inpatient care, hospital teams hand over responsibility for patients to their primary care teams (eg, general practitioners (GPs) or pharmacists) or community care teams (such as community nurses), who deliver care mainly in people’s homes, but also community hospitals or intermediate care facilities. Up to 23% of patients are estimated to experience a safety incident during or soon after discharge, ranging from minor avoidable symptoms to some extreme instances of death, making this period between care settings a vulnerable time for patients.^4^ The safety of patient transitions between care settings is underpinned by myriad behaviours of healthcare professionals (HCPs) involved in patient discharge, such as doctors, nurses and pharmacists. A recent consensus study found that primary care teams consider poor-quality handover instructions, such as those found in discharge documentation such as discharge summaries (DSs) prepared by junior doctors or in district nurse referrals prepared by nurses, to be the most important threat to patient safety during discharge.^2^ The components of poor quality handover instructions were reported to be: missing key information; poor quality of written content, for example, conflicting or incorrect information, instructions still in draft format; or unclear information about medications, therapeutic adjuncts or care equipment required, or follow-up arrangements and referrals.

Discharge documentation enables continuity of care for patients transitioning from hospital to primary care settings. It contains important information for primary or community care teams^5 6^ including patient demographics, registered GP practice, relevant social history, as well as reasons for admission, treatment received, allergies, patient and carer perspectives and details of future care plans.^7^ However, there are no easily available national guidelines on how to prepare DSs. Furthermore, following discharge, there is usually no collaboration between primary/community and secondary care regarding the care of the patient. While previous literature has extensively described deficiencies in discharge documentation,^6 8^ research exploring what factors might lead to such deficiencies and influence preparations of discharge documentation (ie, barriers and enablers) is limited. Furthermore, existing studies^9^,^15^ have only sought the perspectives of doctors, excluding the views of other HCPs involved in preparing discharge documentation (eg, nurses, physiotherapists, occupational therapists) from their evaluations.

Preparing discharge documentation by hospital teams is one of many HCP behaviours necessary to comprehensively hand over care from the hospital to primary care teams. An approach underpinned by behavioural science could therefore generate new insights into why written handovers for primary care teams are considered suboptimal. Behavioural science has been attracting increasing attention in relation to improving health and healthcare, but is rarely translated into policy and routine practice. This has been recognised by the WHO: their 76th World Health Assembly presented the first-ever global resolution on behavioural sciences in 2023, including recommendations for the establishment of behavioural science units and teams within healthcare systems to support the use of behavioural science to address healthcare challenges.^16^

The aim of this study was to systematically identify and explore important barriers and enablers to the preparation of high-quality discharge documentation by HCPs for primary care teams at patient discharge. In order to investigate barriers and enablers in a systematic way, we applied a behavioural science framework, the theoretical domains framework (TDF), to explore influences on the preparation of discharge documentation. The TDF synthesises constructs from 33 behaviour change theories^17 18^ into 14 domains representing cognitive, affective, social and environmental factors influencing behaviours (table 1). The framework was developed to facilitate the identification and labelling of influences on behaviours and to enable subsequent identification of behaviour change strategies to address barriers and enablers to change. This systematic approach facilitates subsequent inclusion of relevant intervention components while excluding ones that might be ineffective against the most important behavioural influences, leading to intervention designs likely to be more effective.^17^,^19^

**Table 1:** The theoretical domains framework (TDF) domains^18^ with corresponding example questions from the study topic guide

Domain of the TDF	Example question from interview topic guide
Knowledge	Do you know of any guidance on how to prepare handover instructions?
Skills	Do you think there are any particular skills required to prepare good quality handover instructions?
Social/professional role and identity	Whose role or responsibility is it to prepare handover instructions for primary care and community teams?
Beliefs about capabilities	How well equipped do you feel you, or your colleagues, are to perform the task of preparing handover instructions for primary care teams?
Optimism	To what extent do you think improving the quality of handover instructions for primary care or community teams has the potential to improve patient outcomes and care?
Beliefs about consequences	What are the consequences of poor-quality handover instructions?
Reinforcement	Are there any rewards or incentives for you or your team to prepare handover instructions to a high standard?
Intentions	To what extent do you ensure that handover instructions are prepared to a high standard?
Goals	How much of a priority is the preparation of handover instructions as a task in your daily workload?
Memory, attention and decision processes	How much effort or attention is required to prepare handover instructions?
Environmental context and resources	What is it about the hospital environment in which you work, or resources available to you, that facilitates or challenges your preparation of handover instructions?
Social influences	To what extent do the views and practices of your colleagues influence how you prepare handover instructions?
Emotion	How do you feel about preparing handover instructions/discharge summaries?
Behavioural regulation	Do you ever discuss or reflect on the quality or process of preparing discharge paperwork with your colleagues?

## Methods

### Context

The study took place in January–April 2021 in clinical and office areas of a large central London teaching hospital, which provides a wide range of elective and emergency, medical and surgical, adult, paediatric and maternity services.

### Participants and sampling strategy

Participants were recruited from different staff groups across medical and surgical wards, representing the range of professionals involved in preparing or contributing to preparations of discharge documentation for primary care teams at patient discharge. A desired sample size of a minimum of 12 participants was selected based on existing literature,^20 21^ including similar studies within the field of patient safety and health services research.^22 23^ Recruitment took place through the research team’s existing professional networks. Junior doctors were recruited in the first instance given they are the main authors of DSs, which form the mainstay of communication between hospital and primary care teams during patient discharge. Nurses, advanced nurse practitioners (ANPs), physiotherapists, occupational therapists and pharmacists were subsequently invited based on discussions with interviewed junior doctors who recommended other HCPs involved in preparing discharge documentation as a routine part of their work. After the first few participants, a snowball technique was adopted to recruit remaining participants. There were no other inclusion or exclusion criteria. Participants received a formal invitation by email to participate. Some participants had a previous professional relationship with the researchers, while others were introduced to the interviewer by email by participants who had already been interviewed.

### Study design and interview guide

Semistructured interviews lasting 45–60 min were conducted in English by one researcher (OMM) via Microsoft Teams or Zoom (based on participant preference) and audio recorded, using institutional accounts which ensure data privacy. Given the timing of the data collection during the COVID-19 pandemic, participants were familiar and comfortable with the online setting. Recordings were transcribed verbatim by a third party and transcripts were anonymised. Transcripts were checked by the researcher against the audio recordings, but not reviewed by participants.

The self-developed interview topic guide was based on the TDF and example questions outlined by Michie *et al*.^24 25^ The interview started with an introduction to the reasons for the interview, namely that the research team was interested in exploring more about how handover instructions are prepared ahead of a patient’s discharge, and what factors may influence the quality of the handover instructions received by primary care and community teams. Questions were initially general and open-ended to understand participants’ clinical roles and roles in patient discharge and preparation of discharge documentation. Participants were then asked a series of fixed open-ended questions covering each domain of the TDF, ensuring comprehensive theoretical coverage and consideration of all potential types of influences. (Not all questions needed to be asked in practice, as participants often spontaneously gave responses which covered multiple questions within the topic guide.) Question-wording was adapted for relevance to each particular staff group (eg, DSs for junior doctors and pharmacists, discharge-to-assess (D2A) forms for physiotherapists and occupational therapists, district nurse referrals for nurses). The topic guide was piloted with a junior doctor to ensure the questions made sense, elicited the desired nature of response and the conversation had a natural flow. Following this pilot interview, minor changes were made to question wording and order. Example questions from the topic guide for each of the 14 domains of the TDF are listed in table 1. The complete topic guide can be found in the online supplemental material (appendix 1). Supplementary notes were made by the researcher following the interviews, capturing the researcher’s reflections to facilitate subsequent analysis.

### Outcomes

The primary outcome was to identify and explore barriers and enablers concerning the preparation of high-quality discharge documentation by hospital staff. The influences likely to be most impactful are described in the text of the results as well as in table 2, categorised according to their corresponding TDF domains. Categorisation by the TDF domain can facilitate the interpretation of findings and support subsequent intervention design.

**Table 2:** Themes, corresponding key influences on preparation of discharge documentation, and illustrative quotes from interview transcripts, per domain of the theoretical domains framework (for the 10 most important influences found in this study)

(B) = Barrier	E= Enabler	(B/E) = Barrier or Enabler
Knowledge		
Theme	Key influence	Illustrative quote
Knowledge of what’s included in discharge documentation	Lack of awareness of guidance or guidelines on how to prepare discharge documentation (B)	“No, I don’t know any guidance. I just seen what the band six and the other nurses are doing and then I just follow. I haven’t seen a guideline or a structure on how to prepare handover.” (Interview 11, Nurse 2)
*Skills*		
Learning how to prepare discharge documentation	Experience writing discharge documentation (E)	“I think I have the skills, but I think it takes a while. It takes a while to kind of get into that mind frame. As I said, I’ve been in this current post for five years, and I take care of a wide variety of therapeutic areas, so anything from HIV to elderly care to hepatology patients, and sometimes – I know the handovers from patient cohort to cohort may change a little bit, and having skills to – and looking at a wide cohort of patient helps in this as well, in terms of the more you do discharges from different cohorts of patients, the more you realise, “Oh okay, this specific group of patients may need something a little bit more than you do.”” (Interview 10, Pharmacist 2)
*Social and professional role and identity*		
Team communication related to discharge documentation	Effective communication within the multidisciplinary team (MDT) (B/E)	“And obviously, if you need to liaise with other people as well to clarify things at times, we need to make appointments maybe with other teams, or another team might need to review things remotely, etc, you know. If we have the contact number at home straight away, can we talk to them via the phone, do they review it straight away or in the next few days, etc, to help prepare that discharge. So, having them at hand rather than fishing through all that information helps as well.” (Pharmacist 2, Interview 10)
*Environmental context and resources*		
The significance of IT in preparing discharge documentation	Software limitations (B)	“Actually, there is a big flaw in the system as well. Once you start a discharge summary, you’ve got the window open in Cerner, you can’t then see anything else in Cerner. So… and you can’t… and so then you’re saving it and then trying to go back, and it’s an absolute nightmare.” (Junior doctor 5, Interview 12)
Physical space where discharge documentation is prepared	Noisy environment in which discharge documentation is prepared, including many distractions (B)	“The noise element actually – we have the radio playing all day. The noise aspect, even though we have so much is actually – you can definitely still – I mean, I wouldn’t say that would affect it. But I think what would affect it is like if the phone’s ringing, if we’re having to speak to relatives, if someone falls.” (Nurse 1, Interview 8)
Organisational and procedural factors	Shift patterns and staff changeovers (B)	“Often we’re writing discharge summaries for patients that we, you know, maybe you’ve seen once or sometimes not at all. I think that is one of the reasons why sometimes maybe they’re not as good as they should be.” (Junior doctor 4, Interview 4)
Time availability for preparing discharge documentation	Availability of time to prepare discharge documentation relative to workload (B/E)	“But I think it will be… the quality is definitely affected by time as well, and when you’re told you need to do a discharge summary but you’re trying to do a million other things at once, you’ve probably rushed it a bit” (Junior doctor 5, Interview 12)
	Workload and volume of tasks more highly prioritised than preparing discharge documentation (B)	“It [a really busy day on a shift] can sometimes compromise what I do write(…)sometimes when it’s really busy, you’ve might get patients mixed up and write the wrong thing on one patient and the other.” (Nurse 2, Interview 11)
*Social influences*		
The influence of hospital colleagues on preparations of discharge documentation	Learning practices from colleagues (B/E)	“I think that the views and practices of the ANPs (advanced nurse practitioners) has a big impact on how I prepare discharge summaries. Because they are the ones I went to for advice early on, when I was learning how to do it. So I think that a lot of what I do is for discharge summaries is sort of learnt from them.” (Junior doctor 4, Interview 4)
Feedback on prepared discharge documentation	Lack of feedback for staff preparing discharge documentation (B)	“I think that feedback is the most important incentive, probably. Because a lot of what me and my colleagues want is just to know we’re doing an okay job. And I think that, knowing that we were or that we weren’t, I mean, that would be the probably the best incentive. Because at the end of the day, we do care about our patients and we care about their outcomes outside of hospital. So knowing it was working would be good.” (Junior doctor 4, Interview 4)

(B)=Barrier; (E)=Enabler; (B/E)=Barrier or Enabler.

### Analysis

Anonymised transcripts were analysed on NVivo V.12 using reflexive thematic analysis as described by Braun and Clarke,^26 27^ as this method encourages the researcher to be reflexive throughout the analysis, supporting the creation of themes portraying the researcher’s interpretation of the qualitative dataset. Analysis followed the six steps of reflexive thematic analysis: familiarisation with the data; generating codes; constructing candidate themes; reviewing potential themes; defining and naming themes; and producing the report.^26^ A mixed inductiv-deductive approach was used with the TDF as a theoretical framework, to group the codes in order to support the construction of themes. First, the lead researcher (OMM) and another researcher (AA) familiarised themselves with one transcript and inductively generated codes, after which codes and meanings within the data were discussed with the wider research team. OMM and AA then repeated this process by independently generating codes for two further transcripts before again meeting to discuss and refine generated codes with the wider research team (GJ, DDL) to resolve discrepancies and confirm accurate representation of the data. OMM then independently generated codes for the remaining transcripts.

**Figure 1:**
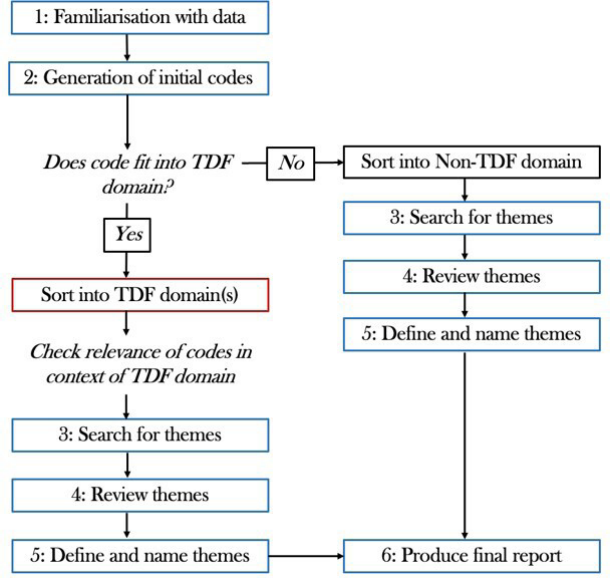
Study coding and theme generation process. The numbered steps reflect those in the process by Braun and Clarke.^26^ TDF, theoretical domains framework.

Once coding was complete, codes were sorted deductively into the 14 TDF domains (OMM and AA). A non-TDF domain was created for codes which did not fit within the TDF. Codes were assigned to more than one domain when necessary. The two researchers first independently allocated codes into TDF domains, and then met to discuss findings and resolve discrepancies with the wider team. After codes were sorted into domains, the codes were reviewed to ensure they still accurately represented the data in the context of the domain in which they were now residing. Within each domain, OMM then inductively grouped codes (eg, *Appreciating time pressures in primary care* and *Thinking about primary care needs when writing discharge documentation*) to create themes (eg, *The influence of primary care and community colleagues*), framed as influences on preparations of discharge documentation. Influences were labelled as enablers, barriers or mixed (eg, if the same influence was a barrier to some participants but an enabler to others). Themes were regularly discussed with the wider research team in order to review and iteratively refine themes, and to define and name themes. Themes could contain multiple influences. Figure 1 shows the analytical process and how the deductive mapping to TDF domains fits between the second and third steps of the reflexive thematic analysis process by Braun and Clarke.^26^

Importance of influences in terms of selecting those that should be targeted in order to improve discharge documentation and patient safety was determined through group discussion using criteria adapted from Atkins *et al*^17^ and Patey *et al*^28^: (a) frequency with which the influence was mentioned, either by one participant or by multiple participants, (b) absence of conflicting beliefs on the nature of the influence, (c) whether the information was volunteered or in response to direct questioning and (d) evidence that influence affects the target behaviour of preparing high-quality discharge documentation.^28^ These criteria formed a basis for discussion rather than strict rules by which to determine the importance of influences, and decisions were made in consensus-based discussions between researchers.

### Researcher characteristics and reflexivity

Interviews were conducted by a female researcher who was a doctor (surgeon) as part of her PhD, throughout which she received training in qualitative data collection. The researcher had a personal interest in improving discharge documentation, having worked as a junior doctor and prepared numerous DSs herself. The researcher consistently discussed findings with the broader research team to mitigate the potential for bias arising from preconceived ideas of influences on how discharge documentation is prepared. The broader research team included clinical and non-clinical academics, including nurses, surgeons and psychologists, who scrutinised interpretations of data and final conclusions.

### Patient and public involvement

Plans for this study were presented to the Imperial Partners Research Group in February 2018 as part of a broader project to improve patient safety during discharge. In November 2020, two patients were invited to review and give feedback on the interview topic guide to ensure the questions remained patient-centric.

## Results

### Participants

12 participants were interviewed (9 female, 3 male), including junior (n=7) and senior (n=5) staff. ‘Senior’ was defined as having been in a role for >5 years or having a leadership role. ‘Junior’ staff were in a role of <5 years at the time of the interview. Participating staff groups included doctors (n=5), an ANP (n=1), nurses (n=2), an occupational therapist (n=1), a physiotherapist (n=1) and pharmacists (n=2), from both medical and surgical specialties. Once participants had agreed to participate, none dropped out of the study.

### Key influences on the preparation of high-quality discharge documentation

44 influences were identified which could all be categorised into a TDF domain. 10 influences were deemed most likely to be important within the following five TDF domains: Knowledge, Skills, Social and professional role and identity, Environmental context and resources and Social influences. Following this prioritisation process, there were six reasons why some influences identified within themes were not considered to be important influences to target in order to improve discharge documentation. The most common reason was that the influence was an existing enabler, and therefore not considered a priority to address in order to facilitate meaningful behaviour change. Other reasons were that influences were: only expressed in response to direct questioning, rather than being volunteered, suggesting that they were not considered to be especially important; only mentioned by, or relevant to a few participants; better described under another TDF domain; not uniquely relevant to preparing discharge documentation; or found to be different in perceived importance between participants. All themes generated within TDF domains, together with their corresponding identified influences, are provided in the online supplemental material (appendix 2). The 10 key influences, categorised by TDF domain, are shown in table 2 and described below.

Outside of the key influences identified, it is important contextual information to note that preparing discharge documentation is a key feature of the regular workload of many different HCPs, and this documentation takes different forms between different professions (eg, DS, D2A, nursing referrals). While the focus of written handovers differs according to professional group (eg, doctors vs physiotherapists), participant responses highlighted substantial overlaps in the information professionals convey in their handovers.

#### Knowledge

##### Lack of awareness of guidance or guidelines on how to prepare discharge documentation

Participants comprehensively described what should be included in discharge documentation, including plans for follow-up, changes to regular medications, pending investigation results, instructions on wound dressings or patients’ rehabilitation goals. However, participants were unaware of formal local, national or international guidelines on best practices related to preparing discharge documentation, relying instead on guidance from their peers, making this a likely important influence on the preparation of discharge documentation.

### Skills

#### Experience writing discharge documentation

Most participants learnt how to prepare discharge documentation on the job, looking to colleagues for guidance (see also the Social influences domain). Experience improved staff comprehension of patients’ conditions and management plans as well as written communication skills, enabling them to succinctly summarise key information for primary care teams through discharge documentation.

### Social and professional role and identity

#### Effective communication within the multidisciplinary team (MDT)

Streamlined two-way communication (ie, contacting and receiving a response)—either face-to-face or through the use of technology such as bleeps, personal mobiles, emails or documentation in electronic health records (EHRs)—enabled preparations of discharge documentation by minimising time spent searching vast patient records for the information required. Effective communication also improved the accuracy of written handovers as it enabled information to be easily clarified with relevant staff members.

### Environmental context and resources

#### Software limitations

Participants uniformly agreed computer software programs used to prepare discharge documentation were complicated to navigate and not user-friendly, and thus an important barrier. Complaints were directed chiefly at the software system used to create DSs, Cerner, where patients’ EHRs were also held. Staff used other programmes such as MS Word or Notes to supplement deficits in Cerner. While participants had doubts as to whether software limitations could easily be addressed, they believed this could lead to tangible improvements in discharge documentation quality.

#### Noisy environment in which discharge documentation is prepared, including many distractions

Discharge documentation was prepared either in wards or clinical offices. While the wards provided proximity to patients as well as professionals looking after them, allowing discharge plans or information to be clarified easily if necessary, the clinical environment was noisy and full of distractions, which negatively impacted preparations for discharge documentation.

#### Shift patterns and staff changeovers

Due to the nature of shift work, staff frequently prepared discharge documentation for patients they had not previously met or did not know well. It was not unusual for a staff member who had been off for a week to be tasked with preparing discharge documentation for a patient admitted while they were away. Furthermore, junior doctors rotating between teams and clinical specialties as often as 3 months meant constantly learning new processes and protocols without being able to fully capitalise on the experience acquired in their previous post if not applicable to their new post. Shift patterns also affected staff working relationships with their colleagues, with subsequent impacts on MDT teamwork and communication.

#### Availability of time to prepare discharge documentation relative to workload

Preparing discharge documentation was described as a time-intensive task which could take up to an hour per patient, with staff often having to prepare discharge documents for multiple patients in a day. Staff uniformly cited ‘having time’, which often they did not, as an enabler in preparing discharge documentation and believed the quality of their written handovers suffered when they felt rushed.

#### Workload and volume of tasks more highly prioritised than preparing discharge documentation

The influence of time availability went hand-in-hand with staff workload, which was frequently cited as a barrier to preparing discharge documentation. Heavy workloads directly influenced time availability, but also distracted staff from focussing on individual tasks and increased the risk of tasks, including preparation of discharge documentation, being rushed.

### Social influences

#### Learning practices from colleagues

Participants described learning how to prepare discharge documentation by observing more experienced colleagues. While the influence of colleagues’ modelling practices was deemed to be an important influence, whether it was an enabler or barrier depended on whether the practices being modelled were according to guidelines.

#### Lack of feedback for staff preparing discharge documentation

Participants reported receiving little or no feedback on discharge documentation prepared by them, from hospital staff, primary care teams, patients or carers. Despite confidence in their abilities to prepare discharge documentation, participants acknowledged this confidence could be misplaced given the lack of confirmation from the recipients of their written handovers, or knowledge of guidelines as to how discharge documentation should be prepared. Participants expressed a desire for feedback not just from primary care but also hospital colleagues, patients and carers, and welcomed intervention ideas involving feedback.

## Discussion

This study systematically explored and identified behavioural influences, and corresponding TDF domains, on the preparation of discharge documentation for primary care teams when patients are discharged from the hospital. Of the 44 influences identified through thematic analysis, 10 were considered most important: Lack of awareness of guidance or guidelines on how to prepare discharge documentation (TDF domain Knowledge); Experience writing discharge documentation (Skills); Effective communication within the MDT (Social and professional role and identity); Software limitations (Environmental context and resources (ECRs)); Noisy environment in which discharge documentation is prepared, including many distractions (ECRs); Shift patterns and staff changeovers (ECRs); Availability of time relative to workload (ECRs); Workload and volume of tasks more highly prioritised than preparing discharge documentation (ECRs); Learning practices from colleagues (Social influences (SIs)); and Lack of feedback for staff preparing discharge documentation (SIs).

The observation that there is substantial overlap between the discharge documentation prepared by different professional groups (eg, DS, D2A, nursing referrals) indicates that there is duplication of work. This represents an important problem in the context of existing staff time limitations and workload, both highlighted here as important influences on preparing discharge documentation.

The ‘Environmental context and resources’ influences of time availability and workload on discharge documentation are consistent with previous evidence that decreasing junior doctors’ workload improves the quality of discharge documentation.^29^ The influence of software limitations on the quality of discharge documentation is pertinent given the ongoing digital transformation of the NHS^30^ and is supported by other studies that highlight some pitfalls of EHRs.^31^,^33^ One systematic review found that EHRs increased the time nurses spent on documentation compared with paper records.^34^ Sarzynski *et al* evaluated the quality of digital DSs and found only 85% contained discharge instructions and many included information irrelevant to postdischarge care.^31^ Participants in our study supported the use of structured headings in discharge documentation, and while this was not considered an important influence in the current behavioural diagnosis, it is an important feature to consider given the high turnover of staff within NHS teams.

The impact of the ‘environment’ in terms of noise and distractions influencing the preparation of discharge documentation is supported by other studies that have explored their impact on staff performance in clinical environments.^35^,^40^ Noise has been found to contribute to staff performance and clinical reasoning in operating theatres and medical errors in wards.^37 38 41^ However, addressing this by providing more dedicated space for staff to undertake administrative duties such as preparing discharge documentation^42 43^ is likely to be challenging.

‘Social influences’, including effective teamwork and communication, were shown to be important enablers for the preparation of discharge documents.^44^ Interventions enabling more effective teamwork and communication in clinical teams, such as visual aids indicating the patient’s readiness for discharge, weekly team meetings or less formal daily huddles were found by Cruz *et al* to improve patient safety and satisfaction during the discharge process.^41^ Input from the MDT enabled generation of comprehensive, holistic discharge plans, but is inextricably linked to the working patterns of hospital staff. Logistical barriers to effective teamwork, such as staff rotas, highlight the need for watertight handover and communication processes between HCPs.

While many interventions to improve discharge documentation have included feedback, the lack of feedback from primary care, hospital colleagues or patients and carers remains an important ‘Social influence’ barrier. Given participants’ confidence in their skills and knowledge of how to prepare discharge documentation, the lack of feedback appears to result in unawareness of the problem with discharge documentation reported by primary care teams. Further, the lack of awareness of guidelines may also contribute to the confidence in their knowledge being unfounded, especially given the findings of our previous study where GPs highlighted that poor quality handover instructions were a problem.^2 45^ Existing feedback interventions have focused on providing feedback from hospital doctors for hospital doctors on DSs, without involving the recipients of these documents—primary care teams and patients.^42 45 46^ Furthermore, interventions have targeted doctors only, excluding other professionals such as nurses, pharmacists, physiotherapists and occupational therapists.^1315 43 47^,^51^

Despite staff experience, identified under the ‘Skills’ domain, being identified as a positive influence on preparations of discharge documentation, DSs are typically authored by the most junior doctors, most of whom have been qualified for less than 1 year.^52 53^ While teaching interventions on DSs for postgraduate doctors is not a new idea,^42 45 46 54^ ^55^ many teaching interventions have focused on the content of the DS rather than signposting guidelines on how to prepare discharge documentation, which would empower healthcare workers to keep their practice up to date.^42 46^

### Limitations

One limitation of this study is that the phrase ‘quality of handover instructions’ was used in the topic guide, however, the term ‘quality’ was not clearly defined for participants. Therefore, they may have defined the term intuitively and may not all have been considering the same standard. While including different HPs involved in preparing different forms of discharge documentation is a strength, the small numbers from different professional groups (and only having one physiotherapist and occupational therapist) is a limitation. However, there were many similarities in influences reported among participants across different professions. Future work could consider exploring differences in influences found between different professional groups.

While recruitment took place via professional networks, there was no coercion or obligation to take part in the study. Despite the clinical role of the main researcher, none of the participants were known to her professionally. Given recruitment from a single hospital, the findings may be restricted by the sample and may not generalise to other settings. The use of a snowball method to support recruitment may also have limited diversity of experience from within the sample.

While the aim of this study was to identify influences on the quality of discharge documentation, the structured approach to data collection based on the TDF is likely to have led to more direct questioning. This may have consequently contributed to influences being mentioned by participants that would not have been otherwise, potentially inflating their importance.^54^ While the topic guide appeared long, several questions were addressed in response to earlier questions, which allowed the interviews to be conducted in a reasonable timeframe. While an approach to thematic analysis involving both deductive and inductive elements is recommended in implementation research,^17^ the process of developing low-level codes (inductive) and then moving them into TDF domains (deductive) before generating themes (inductive) is not a recognised or validated one.^54^ The number of participants recruited was fewer than planned, mainly due to the COVID-19 pandemic, which halted the study for 4 months (January–April 2021). While recruitment finished for pragmatic reasons, the relatively small sample size may be considered sufficient given informational power, as the high amount of relevant information in the sample means that fewer participants are needed.^56^ It also appeared that new information was not being generated after the ninth interview, although data saturation is not an appropriate criterion for determining sample size in reflexive thematic analysis.^57^ Finally, the findings of this study were not presented to participants for feedback, which would have further strengthened the credibility of the conclusions.

### Implications

To optimise patient safety during transitions between care settings, the problem of poor-quality handover instructions in discharge documentation for primary care teams must be addressed, together with the diverse contextual, social, skill-based and knowledge-based influences on their preparation. Identification of the most important TDF domains in the context of preparing discharge documentation facilitates subsequent identification of suitable intervention components, which could be delivered in myriad ways (online supplemental material (appendix 3)) resulting in theory-based intervention design.^23 25 32^ Interventions to improve discharge documentation should target influences identified in the present study but strive to make the task easier and less time intensive. At a time when the NHS is under great pressure, leaders should be prepared to either divert or ring-fence more time for the discharge process to transition patients into the community efficiently and safely, to reduce the risk of avoidable patient harm and consequent unnecessary expenditure resulting from hospital readmissions. However, the suggestion that HCPs invest more time on discharge processes, including preparing DS, must be offset against other responsibilities such as tending to acutely unwell patients, and intervention designers must be mindful of any trade-offs which may consequently occur. Therefore, interventions to improve discharge documentation should target influences identified in the present study while striving to make the task easier and less time intensive. The disparate forms of discharge documentation for primary care challenge intervention designers to separately target each type in improvement initiatives and to consider the contributions of professionals other than doctors who are involved in preparing discharge documentation. We recommend that efforts should focus on MDT collaboration and streamlining various forms of discharge documentation to improve the comprehensiveness of written handovers to primary care without overwhelming community teams with unnecessary information and minimising the time required of hospital HCPs for their preparation.

To overcome the barriers of lack of awareness of relevant guidelines and experience, medical leaders should leverage the experience of more senior staff and consider how junior doctors and other professionals involved in preparing discharge documentation can gain experience through existing teaching opportunities or as undergraduate clinical students. Future interventions should involve feedback from primary care teams, signposting to current, user-friendly best-practice guidelines and regular opportunities to practice the skill of preparing discharge documents.

## Conclusion

This study identified 10 likely important influences on the preparation of high-quality discharge documentation by hospital staff for primary care teams, across five TDF domains. Important factors included: individual factors such as skills in writing DSs, and knowledge of guidelines or what should be included; social factors including effective communication between professionals, learning from others and getting feedback; and environmental influences including noisy environments, software limitations and challenges, high workload and staff changeovers leading to unfamiliarity with the patient. These influences represent potential targets for intervention designers to improve handovers between hospital and primary care and patient safety at discharge. This study demonstrated that preparation of handover documentation is a complex process, requiring multiple actors and different systems, and often takes place in busy, time-pressured environments. Interventions designed to improve the quality of handover documentation should recognise that complexity. Professional and public stakeholders should be involved in the intervention design to ensure it can work in real hospital settings and be effectively incorporated into clinical practice.

## Supplementary material

10.1136/bmjopen-2024-090753 online supplemental file 1

## Data Availability

Data are available upon reasonable request.
