# Supplementary material for: What influences preparations of discharge documentation at patient discharge? An interview study with hospital health professionals based on the theoretical domains framework
Source: BMJ Open. 2025 Jun 16;15(6):e090753. doi: 10.1136/bmjopen-2024-090753 (PMC12314826; doi:10.1136/bmjopen-2024-090753)
Supplement: online supplemental file 1 [file bmjopen-15-6-s001.docx]

**SUPPLEMENTARY MATERIAL**

**Appendix 1: Interview Topic Guide**

**Introduction**

Thank you for taking the time to talk with me today.

- The overall aim of this interview is to understand more about how **handover instructions for primary care and community teams** are prepared ahead of a patient’s discharge home**.**
- We want to know **when and how handover instructions are prepared** and **what factors may influence the quality** of the handover instructions received by primary care and community teams.
- There are no right or wrong answers to any of the questions, and your responses will remain anonymous.
- I would like you to feel comfortable saying what you really think and feel. Please feel free to skip over any questions you do not wish to answer, and to answer in as much or as little detail as you would like.
- Please let me know if you would like to take a break or stop the interview at any point.

Questions to be asked to all participants are in **bold.** Prompts are in *italics*, to be asked if and when needed (e.g. if participants do not understand a question or to help encourage further discussion).

Before starting the interview, ensure the participant has:

1. Received and understood the information sheet
2. Had an opportunity to ask questions
3. Signed the consent form

If the participant has consented, switch the recorder on.

| **GENERAL OPENING QUESTIONS** | | |
| --- | --- | --- |
| **1** | **Can you start by telling me a little bit about yourself:**   - *What is your current professional role and grade?* - *How long have you worked at this trust and department?* | General |
| **2** | **Can you think back to the last time a patient under your care was discharged, or the last time you were involved with a patient’s discharge. Can you talk me through what was done to prepare for their discharge?**   - *When were these preparations made? And by whom?* | General |
| **3** | **What do you understand by the term “handover instructions from hospital to primary care teams?”**   - *Are “handover instructions for primary care and community teams” present anywhere other than discharge summaries? If so, where?* - *When were these prepared?* - *What information did they contain?* - *Who was involved in preparing the handover instructions?* - *How were they sent to primary care or community teams, and by whom?* - *Did you encounter any challenges when preparing the handover instructions/discharge summary? Anything that helped overcome these challenges?* - *How are handover instructions prepared in your department? E.g. paper, computer, on Cerner.* | General |
| **4** | **What is your role in the preparation of handover instructions for primary care and community teams?**   - *If relevant: why is it not your role?* | General |
| **THEORETICAL DOMAINS FRAMEWORK**  **I am now going to ask some specific questions to explore factors that may influence how handover instructions for primary care and community teams are prepared in anticipation of a patient’s discharge from hospital.** | | |
| **5** | **Do you know of any guidance on how to prepare handover instructions?**   - *E.g. content of discharge summaries, information required by primary care and community teams for safe continuity of care* - *Are these guidelines, policies or protocols international, national, local hospital or departmental?* - *What do these guidelines recommend?* | Knowledge |
| **6** | **What information do you need about a patient to prepare good quality handover instructions?**   - *Where do you find/get this knowledge?* - *How readily available is this information? Are there any challenges to accessing this information?* | Knowledge |
| **7** | **What information about patients do you think should be included in handover instructions for primary care teams?**   - *What constitutes “good” handover instructions in your opinion?* | Knowledge / Memory, attention and decision processes |
| **8** | **How do you decide what information to include in handover instructions?**  **Do you always do this?**  **What enables or prevents you from doing this?** | Memory, attention and decision processes |
| **9** | **Do you think there are any particular skills required to prepare good quality handover instructions?**   - *For example communication, writing, interpersonal skills etc?* | Skills |
| **10** | **To what extent do you think you have these skills?**   - *If applicable: how confident are you in using the computer software programmes required for generating handover instructions/discharge summaries?* | Skills |
| **11** | **Can you describe what training or teaching you have had on how to prepare handover instructions for primary care and community teams?**   - *Who delivered this training? (profession/specialty, grade/seniority)* - *When is it or was it delivered? If applicable: have you had any training since?* | Skills |
| **12** | **How well equipped do you feel you, or your colleagues, are to perform the task of preparing handover instructions for primary care teams?**  Are there **any particular patient groups** for whom you, or your colleagues, find it a challenge to prepare discharge summaries?  **What, if any, are the additional challenges associated with preparing handover instructions for:**   - **Elderly patients** - **Patients with complex medical problems taking >5 medications** - **Patients being discharged on anticoagulants?**   **How confident** **are you** **that you, or your colleagues, are able to produce good quality discharge summaries?**   - *Low confidence:* What prevents you from feeling confident? What would make you more confident? - *High confidence:* What enables you to do this task well? | Beliefs about capabilities |
| **13** | **Where do you prepare your handover instructions?**   - *Location: ward, office* - *Computer or paper, specific software programme?* | Environmental context and resources |
| **14** | **Is there a dedicated document, form or software programme where you write handover instructions?**   - *Is there a structured template?* - *How easy is it to use?* - *Do you think the format/boxes are appropriate for how you want to write handover instructions? Is there anything missing? How could it be improved?* | Environmental context and resources |
| **15** | **How familiar do you need to be with your physical working environment and its resources in order to effectively prepare handover instructions for primary care teams?**   - *E.g. the computers, software programmes, location of forms on hard drive or readily available hard copies?* - *What information do you need about your working environment to enable you to effectively prepare handover instructions for primary care teams?* | Environmental context and resources |
| **16** | **What are the barriers and facilitators to gaining the necessary familiarity with local resources and environment when preparing patients for discharge?**  *E.g. does this get covered in your hospital induction, or do you find this out from your colleagues, by yourself while on the job*? | Environmental context and resources |
| **17** | **What is it about the hospital environment in which you work, or resources available to you, that facilitates or challenges your preparation of handover instructions?**  *To what extent do you have enough:*   - *Time to prepare handover instructions, any protected time?* - *Access to the necessary information or resources (computers, space, logins etc*) | Environmental context and resources |
| **18** | **(From ethnography)**  **Whilst observing staff performing routine ward work during a different study, I noted that patient notes and documentation are now mostly digital. Have you encountered any difficulties as a result of this transition from paper to digital notes?**   - *What are the pros and cons of using digital platforms in the clinical setting, both in general and in preparing handover instructions?*   **I also noticed that despite the transition of documentation to digital platforms, many professionals still use paper lists. Do you still use paper lists or notes day to day? What for and why?**  **Data from this study also showed that multiple computers are often taken on ward rounds, drug rounds or into meetings. Do you do this, or have you seen others do this? What are the reasons for this?** | Environmental context and resources |
| **19** | **Do you think the practice of preparing handover instructions varies across wards or specialties** *(are some teams better or more efficient at preparing handover instructions than others)?* **How so?**   - *Is there any sharing of best practice between teams? If relevant: why not?*   **What do you think influences these differences in practices between teams?**   - *Social influences i.e. team dynamics, ward culture, leadership, presence of colleagues on ward or lack of.* - *Physical aspects of clinical environment in which teams work e.g. access to computers, whether team based in a single ward or across multiple sites in hospital, dedicated area for administrative work, clinical schedule.* | Social influences */* Environmental context and resources |
| **20** | **What is it about the social environment in which you work that facilitates or challenges your preparation of handover instructions?**  *By social I mean* *teamwork, workplace culture, work schedule, work hours etc* | Social influences |
| **21** | **Whose role or responsibility is it to prepare handover instructions for primary care and community teams?**   - *Profession? Grade?* - *Why them?* - *Whose role or responsibility do you think it should be?* | Social/ professional role and identity |
| **22** | **Do you and your colleagues work as a team to prepare handover instructions? How?**  *Are there any challenges?* | Social influences |
| **23** | If relevant: **Do you feel supported by your colleagues when preparing handover instructions for patients?**  If relevant**: To what extent do you as a nurse/pharmacist/more senior doctor support colleagues in preparing handover instructions? What helps with this? What are the challenges?** | Social influences |
| **24** | **To what extent do the views and practices of your colleagues influence how you prepare handover instructions? How?** | Social influences |
| **25** | **Do you feel pressure from any of your colleagues when preparing handover instructions? In what way? How does this influence your preparations?** | Social influences |
| **26** | **Do you feel pressure from primary care or community teams when preparing handover instructions? In what way? How does this influence your preparations?** | Social influences |
| **27** | **To what extent do patients and/or their relatives influence your preparations of handover instructions? How?**  *To what extent does your familiarity with a patient influence your preparations of handover instructions about their care?* | Social influences |
| **28** | **What could be done to better support teamwork and communication around preparing handover instructions?** | Social influences |
| **29** | **What do you hope to achieve through preparing handover instructions:**   1. **in the short term (hours-days after patient discharge)?** 2. **In the long term longer term (weeks-months after patient discharge)?** | Goals |
| **30** | **What are the consequences of well-prepared handover instructions?**   - *For primary care teams* - *For patients* - *For you?* | Beliefs about consequences |
| **31** | **What are the consequences of poor-quality handover instructions?**   - *For primary care teams* - *For patients* - *For you?* | Beliefs about consequences |
| **32** | **Do you ever think about these potential consequences when preparing handover instructions?**  **If so, do you think it affects how you prepare handover instructions?** | Beliefs about consequences |
| **33** | **How much effort or attention is required to prepare handover instructions?** | Memory, attention and decision processes |
| **34** | **Do you ever forget to prepare handover instructions? Or forget to include certain information?** | Memory, attention and decision processes |
| **35** | **Do you have any strategies for approaching the task of preparing handover instructions/discharge summaries?**   - *Anything you need to do before?* - *Anything that can help prompt you to do it?* | Behavioural regulation |
| **36** | **Do you ever get feedback on handover instructions?**  *E.g. from primary care, patients, other colleagues in secondary care etc?* | Behavioural regulation |
| **37** | **Do you ever discuss or reflect on the quality or process of preparing discharge paperwork with your colleagues?** | Behavioural regulation |
| **38** | **How do you overcome any barriers or challenges you encounter while preparing handover instructions?**  ***If applicable:* How has covid-19 impacted your/the preparation of handover instructions?** | Behavioural regulation |
| **39** | Clinicians work can sometimes generate a range of emotions and feelings, both positive and negative.  **How do you feel about preparing handover instructions/discharge summaries?**   - *For example do you look forward to it? Or dread it?* | Emotion |
| **40** | **What are your feelings at the time of preparing handover instructions/discharge summaries? Why?**   - *For example do you ever feel annoyed, overwhelmed, motivated or a sense of satisfaction?* - *Does your emotional state ever affect your preparation of handover instructions? How?* | Emotion |
| **41** | **(From ethnography)**  **How much of a priority is the preparation of handover instructions as a task in your daily workload?**  **What other competing commitments prevent you, or your colleagues, from preparing handover instructions?**  **How do these competing commitments impact your preparation of handover instructions?** | Goals |
| **42** | **Are there any rewards or incentives for you or your team to prepare handover instructions to a high standard?** | Reinforcement |
| **43** | **Are there any punitive measures for you or your team that might influence how handover instructions are prepared?** | Reinforcement |
| **44** | **To what extent do you ensure that handover instructions are prepared to a high standard?** | Intentions |
| **45** | **Do you think the quality of handover instructions for primary care teams can be improved?** | General / Optimism |
| **46** | **Do you think there is a need to improve the quality of handover instructions in this hospital/team/you personally? Do you think efforts should be made to improve the quality of handover instructions from hospital to primary care teams?**   - *To what extent is this something you plan to work on?* | Intentions |
| **47** | **To what extent do you think improving the quality of handover instructions for primary care or community teams has the potential to improve patient outcomes and care? How so?** | Optimism |
| **48** | **Do you anticipate any barriers to trying to improve the quality of handover instructions?** | General |
| **49** | **Do you have any ideas about how the quality of handover instructions can be improved?**  *Do you think you or your colleagues would benefit from:*   1. *Feedback from colleagues (primary or secondary care) or patients and relatives* 2. *Better teaching and training on how to prepare discharge summaries* 3. *More user-orientated software* 4. *Standardised discharge summary proformas* 5. *Incentives* | General |
| **CLOSING QUESTIONS** | | |
| **50** | **Is there anything else related to what we have talked about that you would like to add?** | General |

Thank you for your time, the interview is now over. (Stop recording)

**Appendix 2: Identified themes and corresponding influences on the preparation of discharge documentation, per domain of the Theoretical Domains Framework**

**Supplementary Table:** Themes and Corresponding Influences on Preparation of Discharge Documentation, Per Domain of the Theoretical Domains Framework. **(B) =** Barrier, **(E) =** Enabler, **(B/E)** = Barrier or Enabler

| **​Knowledge** | | |
| --- | --- | --- |
| **Theme** | **​Corresponding Influence(s)** | **Supporting quote** |
| ​Knowledge of what’s included in discharge documentation | ​Knowledge of what to include in discharge documentation **(E)** | *Why they came in, any key investigations that have found something or ruled out something, which I think is important […] Anything we did, how we treated it, what we did, were they on antibiotics, any new allergies […] And then a two, three point plan as to what we want them to do, for example, the patient to complete a course of antibiotics, needs follow up blood testing in two, three weeks.*  Interview 12, Junior doctor 5 |
|  | ​Lack of awareness of guidance or guidelines on how to prepare discharge documentation **(B)** | *I just see what the band six and the other nurses are doing and then I just follow. I haven't seen guidelines or a structure on how to prepare a handover.*  Interview 11, Nurse 2 |
| ​Knowledge of local environment and its relevance to preparing high quality discharge documentation for primary care teams. | ​Knowledge and understanding the working environment related to discharge documentation. **(E)** | *I will try to go to the notes and look for the endoscopy report. If I cannot find it then I actually go into the Scorpio software which is used by the endoscopy unit where they log all the different endoscopies for the patients and then I would try to search from there I can retrieve the information. So you need to have, I guess, skill and access to know where to look for the information.*  Interview 2, Junior doctor 2 |
|  | ​Lack of adequate inductions into the working environment **(B)** | *I am involved in some doctors induction and for pharmacy and medication it is 45 minutes that you have got to cram in all the information that they need. It is not enough. I am not doing Cerner in that, thank God. But I think the Cerner they get in that session, within that teaching session, is only about an hour. It is not enough.*  Interview 9, Pharmacist 1 |
|  | ​Staff feeling unsure about local processes **(B)** | *Any social care staff involved with TDS* [three times per day] *package of care and arranging home nursing and home, yeah, so anything that happens at home […] I guess there's a challenge in knowing who arranges it. Is it secondary care or primary care? As I said, I'm not sure.*  Interview 3, Junior doctor 3 |
| **​Skills** | | |
| ​Good communication skills required to prepare high quality discharge documentation | ​Possession of good written and verbal communication skills **(E)** | *You need to be able to hand over valid, reasonable information to the primary care so that you can take care of the patient, if you don't have good communication skills, you're likely to miss out vital information that you should have handed over, which then can lead to that primary carer finding out by themselves and not feeling very confident.*  Interview 11, Nurse 2 |
| ​Learning how to prepare discharge documentation | ​Experience writing discharge documentation **(E)** | *The more you do discharges from different cohorts of patients, the more you realise, “Oh okay, this specific group of patients may need something a little bit more than you do.”*  Interview 10, Pharmacist 2 |
|  | ​Lack of formal training on how to prepare discharge documentation **(B)** | *But there hasn't been any update on training, which would probably be something that […] would be something good to do.*  Interview 6, Physiotherapist |
| **​Social and Professional Role and Identity** | | |
| ​Preparing high quality discharge documentation is reflective of professional identity | ​Sense of pride and responsibility for content of discharge documentation **(E)** | *For me, it’s an art, you know. I’m in this role because I want to help with that. I want to help with having excellent handovers from primary care to secondary care, and to improve that all around the board, you know, from the whole hospital, to my team, to myself. I enjoy it, you know. I might be the odd one out, but I enjoy it.*  Interview 10, Pharmacist 2 |
| ​Impact of team structures on preparation of discharge  ​documentation | ​Fixed and clear team structures **(E)** | *So a firm structure is where an F1 is allocated to a team, which is fixed for the entire rotation […] Really, as a team they will handle an on call together […] In that instance, the F1 is really well suited to dealing with the discharge because although they haven't really made any decisions, they're the ones who've been directed to do all the different things required for patients. […] And I think that makes perfect sense that they are the ones to do the discharge*  Interview 4, Junior doctor 4 |
|  | ​Presence of a dedicated surgical liaison consultant/ registrar who can provide advice relevant to preparing discharge documentation **(E)** | *I think where you have very good surgical liaison teams... and actually […*] *we're very lucky. We do have some very... the ortho-geriatricians are great, and I think... and there is definitely quite a lot of respect between the two teams, surgical liaison and then the surgeons. I think it works well.*  Interview 12, Junior doctor 5 |
| ​Team communication related to discharge documentation | ​Effective communication within the MDT **(E)** | *And obviously, if you need to liaise with other people as well to clarify things at times, we need to make appointments maybe with other teams, or another team might need to review things remotely, etc, you know. If we have the contact number at home straight away, can we talk to them via the phone, do they review it straight away or in the next few days, etc, to help prepare that discharge.*  Interview 10, Pharmacist 2 |
| **​Beliefs About Capabilities** | | |
| ​Confidence in own skills and ability | ​Confidence in own skills and ability to prepare high quality discharge documentation **(B/E)** | *I think, on our ward, given that we've had this discharge summary meeting with our consultant weekly, it makes me quite confident that me and my colleagues and my SHO* [senior house officer] *on our ward can do them quite well.*  Interview 1, Junior doctor 1 |
| ​Confidence using resources required for preparing discharge documentation | ​Confidence using resources required for preparing discharge documentation **(E)** | *No, I don’t think that’s a factor. I mean, we always have computers there, etc, and we know what programmes to use to effectively do what we do*  Interview 10, Pharmacist 2 |
| **​Optimism** | | |
| ​Improving discharge documentation is valuable and  ​feasible | ​Feeling discharge documentation can be improved **(E)** | *I definitely think that there is always room for improvement.*  Interview 1, Junior doctor 1 |
|  | ​Feeling discharge documentation can improve patient care **(E)** | *I think the quality improvement projects are a good way to do it, because that helps to motivate people to get a QIP* [quality improvement project] *done, but also is improving patient care.*  Interview 1, Junior doctor 1 |
| **​Beliefs About Consequences** | | |
| ​Consequences for primary care teams | ​Understanding consequences for primary care teams of both poor and good quality discharge documentation **(E)** | *They're more likely to not have a safe return to the community. So, follow up and prescriptions and further investigations being missed. And yeah, again, the flipside, so a much more difficult job for the GP to work out what's going on.*  Interview 4, Junior doctor 4 |
| ​Consequences for patients | ​Hospital staff thinking about patient welfare and outcomes when preparing discharge documentation **(E)** | *Often, if forms are written badly, it might be that something pivotal is missed. For instance, they might not have adequate or pressure area management, they might be sent home without the receiver acknowledging that they need thickened fluids. They might be sent home without what we call Telecare, which is like a pendant alarm or fall sensors. And they might be then, if they had a fall, they might be on the floor for a long time, which can be fatal.*  Interview 6, Physiotherapist |
| ​Consequences for hospital staff  **​** | ​Understanding consequences for hospital staff of both poor and good quality discharge documentation **(E)** | *Because we are a big team and we're very integrated. And because of Cerner […] there's a lot of colleagues who are then seeing that same patient looking at your notes. Also, your MDT colleagues, it makes you think, right, somebody is going to be reading my note and they need to understand my rationale and my thought processes.*  Interview 6, Physiotherapist |
|  | ​Personal satisfaction and sense of pride as a consequence of preparing high quality discharge documentation **(E)** | *I think it's just the satisfaction of knowing that the patient is getting the care that they need and it's going to continue in the community, because I've given sort of an accurate representation of what's happened.*  Interview 1, Junior doctor 1 |
| **​Goals** | | |
| ​Variation in how discharge  ​paperwork is prioritised as a task | ​Variation in how discharge paperwork is prioritised as a task **(B/E)** | *I just tell the patient they need to wait. I'm not going to just do a rubbish discharge summary because, you know, they might not place much value on it, but I can see the importance of it.*  Interview 5, ANP  *Well, it's important that it has to be done, but I wouldn't put it over seeing a ... if I needed to see a patient who was unwell, I would leave the admin to the end. A discharge summary is more of a chronic or it's not an acute issue that needs to be done.*  Interview 3, Junior doctor 3 |
| ​Improving discharge documentation | ​Supporting improvements for how discharge documentation is prepared (processes and quality) **(E)** | *I think you have to balance it* [improving discharge summaries] *though don't you about everything else that's going on … but it certainly would do no harm to improve the quality of our discharge summaries.”*  Interview 5, ANP |
| **​Memory, Attention and Decision Processes** | | |
| ​Preparing discharge documentation requires focus and concentration | ​Lack of focus on task of preparing discharge documentation due to distractions **(B)** | *It tends to be a quick scan of the notes, what they presented with, what we did, any big treatments, any major results. I wouldn't necessarily include all the scans or all the blood results because I think that's a bit of information overload and trying to decipher what the GP needs to know can be tricky […] If I remembered, I would always say any medications we'd changed, which is sometimes quite difficult because there are so many medication changes, but trying to relay those to the GP is important, I know*  Interview 12, Junior doctor 5 |
| **​Environmental Context and Resources** | | |
| ​The significance of IT in preparing discharge documentation | ​Software limitations **(B)** | *Actually, there is a big flaw in the system as well. Once you start a discharge summary, you've got the window open in Cerner, you can't then see anything else in Cerner. So... and you can't... and so then you're saving it and then trying to go back, and it's an absolute nightmare.*  Interview 12, Junior doctor 5 |
|  | ​Limited availability of functioning computers **(B)** | *Availability of computers on the wards, because it is better to do the discharge at the ward level because if you have got a query, you have got the nurse looking after the patient, you have got the patient themselves, you have got access to their medication if you just want to have a look and see. But if the computers are too slow and not working, or everybody is on them, which can be the point depending on the time you are doing the discharge.*  Interview 9, Pharmacist 1 |
|  | ​Accessibility to patient records **(E)** | *Well, as I said, all the information is on the same platform as you create the discharge summary. So that's not usually an issue.*  Interview 3, Junior doctor 3 |
|  | ​Quality of documentation in patient records **(B/E)** | *So I tend to find what I can on our Cerner note system about their sort of impressions or their summary of what their concerns are, and I tend to often copy and paste that information on to the document and just put when that was documented or who that was documented by so that they can then follow that up if there's any more information they want to know.*  Interview 7, Occupational therapist |
|  | ​Clarity of information in electronic health records **(E)** | *So I think what's good is that you don't have to write everything out, which can be difficult if people don't have legible handwriting that's quite clear.*  Interview 1, Junior doctor 1 |
|  | ​Structured template on forms with headings outlining information to be inputted by person completing discharge paperwork **(E)** | *I think the main thing that strikes me is that there's a huge variability depending on the discharge summary and discharge summaries are completed in very different ways. So it may be easier if there is a standardised way of doing those handovers that would make it easier for GPs to understand and sort of have a have a more consistent way of being handed over to.*  Interview 1, Junior doctor 1 |
|  | ​Mandatory fields that must be completed without which discharge documentation cannot be completed or submitted. **(E)** | *[…] the project found that, you know, a lot of patients were getting missed in terms of follow-ups and indications, things like that. Then we mandated that indication and duration of an anticoagulant into Cerner, so the patient cannot be discharged without that information being there, and that helps. So, I think like mandating essential stuff that needs to be on it definitely helps, rather than having a guide and saying, “Guys, you should do this,” because then you’re free in terms of your interpretation. However, if it was mandated, you can’t discharge the patient without that essential information that we’ve discussed as healthcare professionals and agreed upon.*  Interview 10, Pharmacist 2 |
|  | ​Headings in structured templates that lead to fields not being filled out appropriately (e.g., left blank, duplication of information, inappropriate information included) **(B/E)** | *With the discharge to assess paperwork it's a basic Word document which I feel is fine, is user friendly. The only thing like I said, would be more specific sort of headings and making it more clear of what they want in there.*  Interview 7, Occupational therapist |
| ​Physical space where discharge  ​documentation is prepared | ​Noisy environment in which discharge documentation is prepared, including many distractions **(B)** | *The noise element actually – we have the radio playing all day. The noise aspect, even though we have so much is actually – you can definitely still – I mean, I wouldn’t say that would affect it. But I think what would affect it is like if the phone’s ringing, if we’re having to speak to relatives, if someone falls.*  Interview 8, Nurse 1 |
| ​Organisational and procedural factors | ​Shift patterns and staff changeovers **(B)** | *Often we're writing discharge summaries for patients that we, you know, maybe you've seen once or sometimes not at all. I think that is one of the reasons why sometimes maybe they're not as good as they should be.*  Interview 4, Junior doctor 4 |
| ​Time availability for preparing  ​discharge documentation | ​Availability of time to prepare discharge documentation relative to workload **(E)** | *But I think it will be... the quality is definitely affected by time as well, and when you're told you need to do a discharge summary but you're trying to do a million other things at once, you've probably rushed it a bit*  Interview 12, Junior doctor 5 |
|  | ​Workload and volume of tasks more highly prioritised than preparing discharge documentation **(B)** | *“It* [a really busy day on a shift] *can sometimes compromise what I do write […] sometimes when it's really busy, you've might get patients mixed up and write the wrong thing on one patient and the other.”*  Interview 11, Nurse 2 |
| **​Social Influences** | | |
| ​The influence of hospital colleagues on preparations of discharge documentation | ​Learning practices from colleagues **(B/E)** | *I think that the views and practices of the ANPs has a big impact on how I prepare discharge summaries. Because they are the ones I went to for advice early on, when I was learning how to do it. So I think that a lot of what I do is for discharge summaries is sort of learnt from them.*  Interview 4, Junior doctor 4 |
|  | ​Sharing practices with colleagues **(B/E)** | *I mean, we discuss like different ways of managing discharges and handovers, and we learn from each other, saying, “If it was written in a certain way, maybe something might not get missed.” Or, “Maybe we don’t need to include that information because it’s confusing, and you’ve already written this information previously, so you don’t need to duplicate your work,” etc.*  Interview 10, Pharmacist 2  *No, I don't think I've ever discussed the discharge or anything.*  Interview 11, Nurse 2 |
|  | ​Supportive colleagues **(E)** | *I think they're* [ANPs] *are always very happy to help and advise on their own experience. So I think that we're pretty well supported in that respect.*  Interview 4, Junior doctor 4 |
|  | ​Placing pressure on hospital colleagues to complete discharge paperwork quickly **(B)** | *Yeah, often I feel a lot of pressure from the nursing staff to discharge the patients. I think patients are often, understandably, very keen to go home and they put pressure on the nurses, then the nurses, in turn, put pressure on us. Which is all very understandable* […] *I think it inevitably makes them* [discharge summaries] *more of a rush job.*  Interview 4, Junior doctor 4 |
|  | ​Different practices or and expectations between teams or specialties **(B/E)** | *I think that other wards are more time pressured, that can lead to discharge summaries being completed over a quicker period of time. So it may not contain as much detail or they may have different ways or templates for completing the discharge summary as well.*  Interview 1, Junior doctor 1 |
| ​The influence of primary care and community teams on preparing discharge documentation | ​Hospital staff considering primary care teams’ needs and challenges. **(E)** | *I have a friend […] who writes essays and they're very, very comprehensive and […] I think, oh, this poor GP has to sit and read this letter […] I think that especially blood results, that might make it easier if you've asked the GP to write a follow up on blood results […] if they know that the sodium was 1.2 when it was on admission, they'd then have a reference point that would have been easier; it would make their life easier.*  Interview 12, Junior doctor 5 |
| ​The influence of patients on preparing discharge documentation | ​Patient expectations and complexity **(B/E)** | *Sometimes they'll request for something in particular to be put on there. So particularly in the Covid Ward, some of them want us to write, for example, that they are out of the isolation period. It's sometimes relevant for housing situations, palliative care, things like that. So we will consider what they are saying. Sometimes there are explanations or requested plans for the GP that patients will want on there. So we'll make sure to include that and always take that into account*  Interview 1, Junior doctor 1 |
| ​Feedback on prepared discharge  ​documentation | ​Lack of feedback for staff preparing discharge documentation **(B)** | *I think that feedback is the most important incentive, probably. Because a lot of what me and my colleagues want is just to know we're doing an okay job. And I think that, knowing that we were or that we weren't, I mean, that would be the probably the best incentive. Because at the end of the day, we do care about our patients and we care about their outcomes outside of hospital. So knowing it was working would be good.*  Interview 4, Junior doctor 4 |
| **​Behavioural Regulation** | | |
| ​Feedback on prepared discharge  ​documentation | ​Giving or receiving feedback on discharge documentation, enabling reflection and changes to practice **(E)** | *Yes, yes, a hundred percent* [feedback] *would be very helpful. Does anyone let us know what we could have added, what we may have done wrong, what we haven't informed the community of. So giving us feedback would really help*  Interview 11, Nurse 2 |

**Appendix 3: Potentially suitable BCTs and suggestions of how they could be delivered to address the most important influences on preparation of discharge documentation**

| **TDF Domain** | **Key influence** | **Example potentially suitable BCTs (*numbered as per BCT Taxonomy*)** | **Example interventions to deliver BCTs** |
| --- | --- | --- | --- |
| ***Knowledge*** | Lack of awareness of guidelines or guidance available on how to prepare discharge documentation | 4.1 Instruction on how to perform behaviour  4.2 Information about antecedents  5.1 Information about health consequences  5.3 Information about social and environmental consequences | 1. Education 2. Easy access to guidebooks 3. Prompts containing instructions |
|  | Uncertainty in how to use and navigate hospital systems, including software programmes and processes |  |  |
| ***Skills*** | Experience of staff in writing discharge documentation | 4.1 Instruction on how to perform behaviour  8.1 Behavioural rehearsal / practice | 1. Education 2. Opportunities to practice preparing discharge documentation 3. Assessments |
| ***Social/professional role and identity*** | Effective communication within MDT | 3.1 Social support (unspecified)  6.2 Social comparison | 1. Creation of “Buddies” within teams to offer support to new or junior colleagues |
| ***Environmental context and resources*** | Software limitations | 3.2 Social support (practical)  7.1 Prompts / cues  10.4 Social reward  12.1 Restructuring the physical environment  12.2 Restructuring the social environment  12.5 Adding objects to the environment | 1. Improvements to or replacement of existing software 2. Redesigning and reformatting documentation and discharge proformas 3. Dedicated quiet spaces for administrative work 4. Hiring more staff 5. Rota adaptations and improvements 6. Provision of additional computers |
|  | Noisy and distracting environment |  |  |
|  | Staff working patterns and team changeovers |  |  |
|  | Availability of time to prepare discharge documentation relative to existing workload |  |  |
|  | Competition for computers |  |  |
| ***Social influences*** | Learning good practices from colleagues | 3.1 Social support (unspecified)  3.2 Social support (practical)  6.2 Social comparison  6.3 Information about others’ approval  10.4 Social reward | 1. Sharing best practices during teaching sessions 2. Giving and receiving feedback (peers, seniors, primary care teams and/or patients and carers) |
|  | Lack of feedback for staff preparing discharge documentation |  |  |
