# Supplementary material for: What influences preparations of discharge documentation at patient discharge? An interview study with hospital health professionals based on the theoretical domains framework
Source: BMJ Open. 2025 Jun 16;15(6):e090753. doi: 10.1136/bmjopen-2024-090753 (PMC12314826; doi:10.1136/bmjopen-2024-090753)
Supplement: Supplementary file 2 [file bmjopen-2024-090753.reviewer_comments.pdf]

PEER REVIEW HISTORY

BMJ Open publishes all reviews undertaken for accepted manuscripts. Reviewers are asked to complete a checklist review form and are provided with free text boxes to elaborate on their assessment. These free text comments are reproduced below.

ARTICLE DETAILS

Title (Provisional)

What Influences Preparations of Discharge Documentation at Patient Discharge? An Interview Study with Hospital Health Professionals Based on the Theoretical Domains Framework

Authors

Markiewicz, Ola Malgorzata; Menon, Aarya; Acharya, Amish; D'Lima, Danielle; Lorencatto, Fabiana; Darzi, Ara; Judah, G

VERSION 1 - REVIEW

**Reviewer**	**1**
**Name**	**Pedersen, Mona**
**Affiliation**	**Aalborg University Hospital, Centre for Clinical Research, North Denmark Regional Hospital**
**Date**	**27-Oct-2024**
**COI**	**None**

Thank you for the opportunity to review this relevant manuscript studying key influences on the preparation of discharge documentation in the transition from hospital to home, and identification of enablers and barriers for the preparation of handover information perceived by the hospital care givers. Especially, it is interesting that you use the behavioural science framework Theoretical Domains Framework as a methodological and theoretical framework. This increases the relevancy and potential transferability of the findings from this specific hospital study setting to other settings and internationally.

The choice of the Theoretical Domains Framework is therefore very relevant, and you offer relevant references of the framework and how it has been validated.

However, there is a need for increasing the linkage between aim, methods, findings, and conclusion.

The following remarks relate to specific sections of the manuscript.

Pg 1-3 Abstract, Strengths and Limitations, Box

Well-written and well-disposed.

Pg. 3-4. Introduction

The aspects included in the introduction are relevant regarding the aim of the manuscript.

I would suggest that you made a short description of the UK health care system and how the hospital collaborates with the primary health care system and whether there are any national or local guidelines for discharge information between hospitals and municipality care. Furthermore, it would be relevant to know how the discharge information process is integrated in the digital transformation and has patients and their family caregivers' access to this information?

It would also be helpful if you specify what it is meant by primary care teams (Pg. 3 line 47-48) Does this also include family doctors? Furthermore, you use the team post-hospital care teams (line 56) and the information that they need (pg. 4 lines 4-6). Do you distinguish between these teams?

The aim of the study is described pg. 4, line 30-31. However, this description differs from the aim described in the abstract – (pg 1 – lines 22-24) and does not correspond with the formulation of Outcomes (Pg 7). Please, consider this when you revise the manuscript.

Pg 4 line 24: Please, use capitals in the abbreviation of World Health Organization

Pg. 4-10 Methods:

Context: (Pg. 5)

Pg. 4 line 53 – You write that the study took place in 2018-2019, which indicate that the data are quite old. Later (Pg. 18 line 17) you write that due to the pandemic the study took place in January-April 2021. Please, clarify these divergencies.

Participants and Sampling strategy (pg. 6)

You describe the recruitment process using first a strategic sampling strategy then a snowball technique initiated by the network of the research team, starting by junior doctors and covered by various professions without further inclusion or exclusion criteria. You ended up with participants from medical and surgical wards (pg. 10 line 26). Was this a part of your sampling strategy or by chance?

Study design and interview-guide (Pg. 5,6,7)

In table 1 you present the TDF domains and illustrate examples of the questions from the interview guide, which gives a fine overview.

However, it remains unclear whether and to which degree the interview questions were self-developed? You also write that the questionnaire was piloted by a junior doctor for clarity, fluidity and comprehension (pg. 6, line 52-53). Please describe how this was provided, the impact hereof and a methodological reference for validation.

You refer to the supplementary material for further details regarding the interview guide. You write that you conducted semi structured interview and the interviews lasted 45-60

minutes (pg. 5 line 33). Based on the supplementary material the interview guide encompasses a total of 50 questions. For me, as a qualitative researcher, it does not seem feasible in a semi structured interview. I will suggest that you either in the methods section or in the discussion, clarifies how the interview guide was used and which influence the number of questions might have had on the interview session (e.g., how did the interviewer prioritize the questions).

Pg. 5, line 33-34 The interviews were conducted via MS Teams – (MS abbreviation for Microsoft??) – or Zoom – please elaborate on how this impacted the interview and how the integrity of data was secured?

Outcomes (pg 7)

Lines 11-14. The formulation of primary outcome deviates from the terminology that was used when you described the aim (e.g. you use the term facilitations instead of enablers). Here, you use the term identify – when you describe the aim (in the abstract) you use the term explore.

Please, align the essential formulations throughout the manuscript to increase the consistency.

Analysis (pg. 8-9)

You describe that the analysis was based on the reflexive thematic analysis by Braun and Clarke (pg. 7, line 22). However, I find neither the rationale for using Braun & Clarke nor methodological references to substantiate the use of this analytical approach.

Figure 1 illustrates the steps in the analysis process without referring to the steps used by Braun and Clarke.

Later (pg. 18, lines 11-15) you describe the inductive and deductive coding process – this description belongs to the method section (pg. 7)

To increase the transparency of your work I would suggest that you refer to the primary literature offered by Braun and Clarke and use their terminology when you describe the analysis process.

To determine the importance of the influences, you refer to the four criteria by Atkins and Patey (pg. 8, lines 31-39). I suggest that you give examples of how these criteria were used in the group of researchers and how they were balanced.

Researcher characteristics and reflexivity (Pg. 8)

Important part of the internal validation of a qualitative study and provides transparency.

You write that a doctor (MBBS) provided the interviews. MBSS is probably the medical degree of the interviewer and not the initials. However, using abbreviations without explaining them can create misunderstandings for the reader.

Another problem in this section is that there is not consistency between singular and plurals – e.g., you write the researcher and refer to their …..

Please rewrite this section for clarification.

Patient and public involvement (Pg. 10)

Pg. 10 line 7-10, you write that two patients reviewed and gave feedback on the interview topic guide November 2020.

I value that patients are involved in research and agree that they can contribute with important perspectives when it comes to initiatives about quality and safety.

However, I would question the relevance of the patient perspective to give feedback on the topic guide. This guide was focusing on barriers and enablers that influenced the preparation process and not on the quality and relevance of the information provided by discharge to the primary care team. What is the purpose of patients' feedback of whether the questions remained patient centric? And what was their response and how did this impact the interview guide?

Pg. 10-16 Results:

Pg 10 line 30-44: You give an overall description of how the key influences were deemed most likely and that existing enablers were not included, because they would probably not generate meaningful behavioural changes if they were targeted. This probably relates to criteria d) (Atkins and Patey, pg 8, line 39) and might be the reason why only one enabler is a part of the ten key influences. However, how did you balance this criterion with the other criteria to assess the importance of influences?

The findings from the analysis are presented in table 2. The table is divided in themes, key influences, supplemented by illustrative quotes, which gives a fine overview.

In addition, each of the ten key influences is described separately in the text, which makes the finding section quite fragmented for the reader. I would suggest that the text was divided into the five domains (Knowledge, Skills, Social and professional Role and Identity, Environmental Context and Resources, Social influences). This would give a more comprehensive presentation and would also be beneficial for the later discussion.

Regarding the table (small details): Please avoid abbreviations that are not explained (e.g, MBT pg. 12, line 11)). Also, be consistent with what is written in bold.

Pg. 16-19 Discussion:

The discussion of the ten key influences is relevant and related to other studies. However, the discussion is difficult to follow, probably due to how the discussion is structured. As already mentioned, the discussion would probably benefit from being structured into the five domains.

Parts of the discussion is not mentioned in the results section – e.g. that there is a substantial overlap (pg. 16; line 49 -56)

Several times you mention challenges for an intervention designer (pg. 17, line 3, line 13, , pg. 18, line 31). It is not clear for me whether this is a part of the discussion and how it relates to the findings/results). Instead, this could be a part of the implications and conclusion.

Limitations (pg. 18, lines 1-24)

You mention the potential pitfalls related to using the TDF both to construct the interview guide and as the analytical tool, which is relevant. Furthermore, it is relevant to discuss the numbers of questions related to the timeframe of the interview.

I need a more thorough description and deeper discussion of the limitations related to some of the methods - e.g. the thematic analysis based on Brown and Clarke. Especially, when you claim that data saturation was reached by the final participants. In regard to saturation and sample size I suggest that you look further into this paper: Braun, V., & Clarke, V. (2021). To saturate or not to saturate? Questioning data saturation as a useful concept for thematic analysis and sample-size rationales. Qualitative research in sport, exercise and health, 13(2), 201-216. It is not sufficient to refer that other studies have used similar sample sizes.

Finally, I would like that you discuss the sampling strategy – benefits and pitfalls – and as a part of this, ethical and data-integrity aspects.

Implications (pg. 18, lines 28-55)

This section lifts the study findings to become more general challenges related to improve the process of handover instructions from hospital to primary health care and increase the transferability of the results. And thereby supports the relevance of using behavioural science and the Theoretical Domains Framework as an analytical tool within the healthcare setting.

I will suggest that you refer to Michie et al 2014, which is already used as a reference.

Conclusion (Pg. 20 lines 1-16)

This conclusion does not add anything new to the aims/research questions of this study – only that you found ten influences across five TDF domains. I suggest that you add more information of the findings: e.g. which domains were found to be important, such as individual factors such as knowledge and skills, professional roles and identity, environmental and contextual factors and social and cultural aspects. (You could use parts of the implications section pg. 18 – lines 30-34).

I think that the conclusion should underline that improving the preparation of the handover instructions is a complex process that involve a lot of various processes and actors and is partly context sensitive and that potential future interventions must recognise this complexity.

**Reviewer**	**2**
**Name**	**Castro-Avila, Ana**
**Affiliation**	**University of York, Health Sciences**
**Date**	**31-Dec-2024**
**COI**	**None**

Thanks for the opportunity to review this manuscript, which reports the findings from 12 interviews exploring factors that influence the preparation of discharge summaries. I quite enjoyed reading this paper. I only have one major issue with it: the definition of a high-quality discharge document.

The first line of the abstract says "poor quality handover", and the conclusion refers to "quality of discharge documentation". If I search for quality, it appears 64 times in the document; it even appears on your definition of outcomes; however, you have not defined the quality of a discharge summary. This is problematic because your interview guide uses the term and, therefore, your participants defined "quality" intuitively without guarantee that everyone is talking about the same standard. For me, the quality of a discharge summary depends on its purpose. It should allow a smooth transition into primary care and avoid readmissions or emergency attendances. The professionals who are probably best placed to judge the adequacy or quality of a discharge summary are those who receive it and have to implement a treatment plan based on the information in the summary.

That said, the purpose of a discharge summary will be different for a PT or OT, and, to the best of my knowledge, there are no standard definitions of an adverse event due to poor quality handover for those professions. Maybe a fall within a week after discharge?

This also links to interviewing different professionals. This adds value, but including only one PT and one OT is problematic. Strictly speaking, these professionals report on different processes, and I find it more difficult to see the value of interviewing a single professional.

There are also a couple of typos that I spotted:

Page 5, lines 25-26: "others were by introduced". Please, delete by

In Table 2, there is a quote from "junior doctor 5, interview 12" and another from "junior doctor 12, interview 12". I think there is a typo for one of them.

Also, I could not find a statement about ethical approval or funding.

I hope the author finds these comments useful.

VERSION 1 - AUTHOR RESPONSE

Responses to Reviewer 1 comments:

There is a need for increasing the linkage between aim, methods, findings, and conclusion.

Edits have been made throughout the text to increase the consistency between the aim and other sections of the paper, as requested.

Pg. 3-4. Introduction
I would suggest that you made a short description of the UK health care system and how the hospital collaborates with the primary health care system and whether there are any national or local guidelines for discharge information between hospitals and municipality care.

The following text has been added to the introduction describe the UK health system and how hospitals collaborate with primary care:

"With the UK National Health Service (NHS) experiencing unprecedented demand for beds there is pressure for staff to discharge patients from hospital sooner (1-3). Care in the NHS can be delivered in hospitals, and inpatient care involves an admission to a hospital bed. Following discharge from inpatient care, hospital teams hand over responsibility for patients to their primary care teams (e.g. General Practitioners (GPs) or pharmacists), or community care teams (such as community nurses, who deliver care mainly in people's homes, but also community hospitals or intermediate care facilities)."

"Discharge documentation enables continuity of care for patients transitioning from hospital to primary care settings. It contains important information … <u>However, there are no easily available national guidelines on how to prepare discharge summaries. Furthermore, following discharge there is usually no collaboration between primary/community and secondary care regarding the care of the patient."</u>

Furthermore, it would be relevant to know how the discharge information process is integrated in the digital transformation and has patients and their family caregivers' access to this information?

There is variation between trusts on how discharge summaries are generated and how they are provided to GPs, patients and caregivers. For example in some trusts, while discharge summaries are sometimes emailed to GPs (when GP email addresses are known), patients will only receive paper copies given to them at discharge, or posted. As there is not a consistent process of integrating discharge information in the digital transformation, this has not been included in the paper.

It would also be helpful if you specify what it is meant by primary care teams (Pg. 3 line 47-48) Does this also include family doctors?

I have clarified that this referred to General Practitioners. We do not have the term family doctors.

Furthermore, you use the team post-hospital care teams (line 56) and the information that they need (pg. 4 lines 4-6). Do you distinguish between these teams?

The *phrase "post-hospital care teams"* has been replaced with *"primary or community care teams"* to clarify.

The aim of the study is described pg. 4, line 30-31. However, this description differs from the aim described in the abstract – (pg 1 – lines 22-24) and does not correspond with the formulation of Outcomes (Pg 7). Please, consider this when you revise the manuscript.

Thank you for drawing this inconsistency to our attention. The sections have been reworded as follows to address this:

Abstract:

"The aim of this study was to systematically identify and explore important barriers and enablers to the preparation of high-quality discharge documentation by healthcare professionals (HCPs) for primary care teams at patient discharge."

Introduction:

"The aim of this study was to systematically identify and explore important barriers and enablers to the preparation of high-quality discharge documentation by healthcare professionals (HCPs) for primary care teams at patient discharge. In order to investigate barriers and enablers in a systematic way, we applied a behavioural science framework, the Theoretical Domains Framework (TDF), to explore influences on the preparation of discharge documentation."

Outcomes:

"The primary outcome was to identify and explore barriers and enablers concerning the preparation of high-quality discharge documentation by hospital staff. The influences likely to be most impactful are highlighted, together with their corresponding TDF domains."

Pg 4 line 24: Please, use capitals in the abbreviation of World Health Organization.

The term "who" here was not meant to be an abbreviation. The text has been amended to clarify.

Pg. 4 line 53 – You write that the study took place in 2018-2019, which indicate that the data are quite old. Later (Pg. 18 line 17) you write that due to the pandemic the study took place in January-April 2021. Please, clarify these divergencies.

The reference to 2018-2019 was an error. This has been corrected to refer to January-April 2021 in both places.

Participants and Sampling strategy (pg. 6)
You describe the recruitment process using first a strategic sampling strategy then a snowball technique initiated by the network of the research team, starting by junior doctors and covered by various professions without further inclusion or exclusion criteria. You ended up with participants from medical and surgical wards (pg. 10 line 26). Was this a part of your sampling strategy or by chance?

This was part of the sampling strategy and has been clarified in the text: *"Participants were recruited from different staff groups* *across medical and surgical wards…"*

Study design and interview-guide (Pg. 5,6,7)
In table 1 you present the TDF domains and illustrate examples of the questions from the interview guide, which gives a fine overview. However, it remains unclear whether and to which degree the interview questions were self-developed?

We have added text to clarify that it was self-developed.

You also write that the questionnaire was piloted by a junior doctor for clarity, fluidity and comprehension (pg. 6, line 52-53). Please describe how this was provided, the impact hereof and a methodological reference for validation.

The following text has been added to clarify:

"The topic guide was piloted with a junior doctor to ensure the questions made sense, elicited the desired nature of response and the conversation had a natural flow. Following this pilot interview, minor changes were made to question wording and order."

You refer to the supplementary material for further details regarding the interview guide. You write that you conducted semi structured interview and the interviews lasted 45-60 minutes (pg. 5 line 33). Based on the supplementary material the interview guide encompasses a total of 50 questions. For me, as a qualitative researcher, it does not seem feasible in a semi structured interview. I will suggest that you either in the methods section or in the discussion, clarifies how the interview guide was used and which influence the number of questions might have had on the interview session (e.g., how did the interviewer prioritize the questions).

Not all questions had to be asked as the answers provided by participants often answered other questions in the same section and domain. Future questions that were covered by participant responses were ticked off as the researcher went along, in order to keep track of domains which were outstanding. The following text was added in the methods section:

"(Not all questions needed to be asked in practice, as participants often spontaneously gave responses which covered multiple questions within the topic guide.)"

Pg. 5, line 33-34 The interviews were conducted via MS Teams – (MS abbreviation for Microsoft??) – or Zoom – please elaborate on how this impacted the interview and how the integrity of data was secured?

The following text has been added/amended to clarify:

"via Microsoft Teams or Zoom (based on participant preference) and audio recorded, using institutional accounts which ensure data privacy. Given the timing of the data collection during the Covid-19 pandemic, participants were familiar and comfortable with the online setting."

Outcomes (pg 7)
Lines 11-14. The formulation of primary outcome deviates from the terminology that was used when you described the aim (e.g. you use the term facilitations instead of enablers). Here, you use the term identify – when you describe the aim (in the abstract) you use the term explore.

Please, align the essential formulations throughout the manuscript to increase the consistency.

The term facilitators has been changed to enablers throughout. The wording of the aim has been changed to: identify and explore. Relevant quotes from the paper are given to the response to the question above about aims.

Analysis (pg. 8-9)
You describe that the analysis was based on the reflexive thematic analysis by Braun and Clarke (pg. 7, line 22). However, I find neither the rationale for using Braun & Clarke nor methodological references to substantiate the use of this analytical approach.

The section of the analysis was edited as follows to explain why this method was selected, and to add relevant references:

"Anonymised transcripts were analysed on NVivo 12 using reflexive thematic analysis by Braun and Clarke (26, 27), as this method encourages the researcher to be reflexive throughout the analysis, supporting the creation of themes portraying the researcher's interpretation of the qualitative dataset. Analysis followed the six steps of reflexive thematic analysis: familiarisation with the data; generating codes; constructing candidate themes; reviewing potential themes; defining and naming themes; and producing the report (26). A mixed inductive/deductive approach was used with the TDF as a theoretical framework, to group the codes in order to support the construction of themes."

Figure 1 illustrates the steps in the analysis process without referring to the steps used by Braun and Clarke.

The following sentence has been added to show how the figure relates to the steps by Braun and Clarke:

"Figure 1 shows the analytical process, and how the deductive mapping to TDF domains fit between the second and third steps of the reflexive thematic analysis process by Braun and Clarke."

Later (pg. 18, lines 11-15) you describe the inductive and deductive coding process – this description belongs to the method section (pg. 7)

This information has already been described in more depth in the methods section. The summary in the limitations section is just discussing a potential limitation in the approach.

To increase the transparency of your work I would suggest that you refer to the primary literature offered by Braun and Clarke and use their terminology when you describe the analysis process.

The wording in the analysis section has been updated to ensure terminology is consistent with the steps described by Braun and Clarke.

To determine the importance of the influences, you refer to the four criteria by Atkins and Patey (pg. 8, lines 31-39). I suggest that you give examples of how these criteria were used in the group of researchers and how they were balanced.

The sentence below was added in the analysis section:

"These criteria formed a basis for discussion rather than strict rules by which to determine the importance of influences, and decisions were made in consensus based discussions between researchers, and decisions were made in consensus-based discussions between researchers*."*

The following section has been added in the results:

"Following this prioritisation process, there were six reasons why some influences identified within themes were not considered to be important influences to target in order to improve discharge documentation. The most common reason was that the influence was an existing enabler, and therefore not considered a priority to address in order to facilitate meaningful behaviour change. Other reasons were that influences were: only expressed in response to direct questioning, rather than being volunteered, suggesting that they were not considered to be especially important; only mentioned by, or relevant to a few participants; better described under another TDF domain; not uniquely relevant to preparing discharge documentation; or found to be different in perceived importance between participants."

Researcher characteristics and reflexivity (Pg. 8)
You write that a doctor (MBBS) provided the interviews. MBSS is probably the medical degree of the interviewer and not the initials. However, using abbreviations without explaining them can create misunderstandings for the reader.
Another problem in this section is that there is not consistency between singular and plurals – e.g., you write the researcher and refer to their …..
Please rewrite this section for clarification.

We have clarified that researcher was a surgeon, and have rewritten the paragraph to avoid using plurals inconsistently.

Patient and public involvement (Pg. 10)
Pg. 10 line 7-10, you write that two patients reviewed and gave feedback on the interview topic guide November 2020.
I value that patients are involved in research and agree that they can contribute with important perspectives when it comes to initiatives about quality and safety. However, I would question the relevance of the patient perspective to give feedback on the topic guide. This guide was focusing on barriers and enablers that influenced the preparation process and not on the quality and relevance of the information provided by discharge to the primary care team. What is the purpose of patients' feedback of whether the questions remained patient centric? And what was their response and how did this impact the interview guide?

Given this was a topic guide to facilitate discussions with HCPs, the involvement of patients was minor. Patients were mainly asked to comment on whether there were any areas important to patients not captured by the questions. Patients also commented on any wording which was ambiguous which was adapted accordingly. They also made suggestions regarding order of questions/domains to aid flow.

Pg. 10-16 Results:
Pg 10 line 30-44: You give an overall description of how the key influences were deemed most likely and that existing enablers were not included, because they would probably not generate meaningful behavioural changes if they were targeted. This

probably relates to criteria d) (Atkins and Patey, pg 8, line 39) and might be the reason why only one enabler is a part of the ten key influences. However, how did you balance this criterion with the other criteria to assess the importance of influences?

All of the four criteria from Atkins and Patey were considered. However, key influences were felt to be those that should be targeted in order to improve the quality of discharge documentation. Therefore existing enablers were not likely to meet criteria d) and so be less likely to be included within the ten key influences. Some text has been added to the methods to clarify this process:

"Importance of influences in terms of <u>selecting those that should be targeted in order to improve</u> discharge documentation and patient safety was determined through group discussion using criteria adapted from Atkins et al."

The findings from the analysis are presented in table 2. The table is divided in themes, key influences, supplemented by illustrative quotes, which gives a fine overview. In addition, each of the ten key influences is described separately in the text, which makes the finding section quite fragmented for the reader. I would suggest that the text was divided into the five domains (Knowledge, Skills, Social and professional Role and Identity, Environmental Context and Resources, Social influences). This would give a more comprehensive presentation and would also be beneficial for the later discussion.

The results section is already structured according to the five TDF domains. We feel that removing the subheadings to the influences is likely to make the results section harder to follow and harder to match with examples from the table. We would prefer to keep the current structure, but can consolidate into themes more for the discussion.

Regarding the table (small details): Please avoid abbreviations that are not explained (e.g, MBT pg. 12, line 11)). Also, be consistent with what is written in bold.

We have made the text in bold consistent. The MDT has been explained just prior – multidisciplinary team.

Pg. 16-19 Discussion:
The discussion of the ten key influences is relevant and related to other studies. However, the discussion is difficult to follow, probably due to how the discussion is structured. As already mentioned, the discussion would probably benefit from being structured into the five domains.
The discussion has been edited to structure according to the TDF domains.

Parts of the discussion is not mentioned in the results section – e.g. that there is a substantial overlap (pg. 16; line 49 -56)

Thank you for highlighting this. The following text has been added in the results:

"Outside of the key influences identified, it is important contextual information to note that preparing discharge documentation is a key feature of the regular workload of many different HCPs, and this documentation takes different forms between different professions (e.g., DS, discharge-to-assess, nursing referrals). While the focus of written handovers differs according to professional group (e.g., doctors vs. PTs), participant responses highlighted substantial overlaps in the information professionals convey in their handovers."

The section in the discussion has been amended as follows:

"The observation that there is substantial overlap between the discharge documentation prepared by different professional groups (e.g., DS, discharge-to-assess, nursing referrals) indicates that there is duplication of work. This represents an important problem in the context of existing staff time limitations and workload, both highlighted here as important influences on preparing discharge documentation."

Several times you mention challenges for an intervention designer (pg. 17, line 3, line 13, , pg. 18, line 31). It is not clear for me whether this is a part of the discussion and how it relates to the findings/results). Instead, this could be a part of the implications and conclusion.

These sections have moved to the implications section, as suggested.

"At a time when the NHS is under great pressure, leaders should be prepared to either divert or ringfence more time for the discharge process to transition patients into the community efficiently and safely, to reduce the risk of avoidable patient harm and consequent unnecessary expenditure resulting from hospital readmissions. However, the suggestion that HCPs invest more time on discharge processes, including preparing DS, must be offset against other responsibilities such as tending to acutely unwell patients, and intervention designers must be mindful of any trade-offs which may consequently occur. Therefore, interventions to improve discharge documentation should target influences identified in the present study while striving to make the task easier and less time intensive. The disparate forms of discharge documentation for primary care, challenges intervention designers to separately target each type in improvement initiatives, and to consider the contributions of professionals other than doctors who are involved in preparing discharge documentation. We recommend that efforts should focus on MDT collaboration and streamlining various forms discharge documentation to improve comprehensiveness of written handovers to primary care without overwhelming community teams with unnecessary information, and minimising time required of hospital HCPs for their preparation."

Limitations (pg. 18, lines 1-24)
You mention the potential pitfalls related to using the TDF both to construct the interview guide and as the analytical tool, which is relevant. Furthermore, it is relevant to discuss the numbers of questions related to the timeframe of the interview.

The question about the length of the interview given the number of questions in the topic guide is addressed in a response above. The following text has been added to the limitations to explain this:

"While the topic guide appeared long, several questions were addressed in responses to earlier questions, which allowed the interviews to be conducted in a reasonable timeframe."

I need a more thorough description and deeper discussion of the limitations related to some of the methods - e.g. the thematic analysis based on Brown and Clarke. Especially, when you claim that data saturation was reached by the final participants. In regard to saturation and sample size I suggest that you look further into this paper: Braun, V., & Clarke, V. (2021). To saturate or not to saturate? Questioning data saturation as a useful concept for thematic analysis and sample-size rationales. Qualitative research in sport, exercise and health, 13(2), 201-216. It is not sufficient to refer that other studies have used similar sample sizes.

Following the ninth interview it appeared that new information was not being reported. However, data saturation was not used as a basis for determining when to stop collecting data, as this was not consistent with the reflexive thematic analysis process used, which relies on the interpretative judgment of the researcher, and therefore the generation of themes is considered to never be truly complete. The following text has been added in the limitations, to replace the text about other studies using similar sample sizes.

"While recruitment finished for pragmatic reasons, the relatively small sample size may be considered sufficient given informational power, as the high amount of relevant information in the sample, means that fewer participants are needed (56). It had also appeared that new information was not being generated after the ninth interview, although data saturation is not an appropriate criteria for determining sample size in reflexive thematic analysis (57)."

Finally, I would like that you discuss the sampling strategy – benefits and pitfalls – and as a part of this, ethical and data-integrity aspects.

The following text has been added in the limitations:

"While recruitment took place via professional networks, there was no coercion or obligation to take part in the study. Despite the clinical role of the main researcher, none of the participants were known to her professionally. Given recruitment from a single hospital, the findings may be restricted by the sample and may not generalise to other settings. The use of a snowball method to support recruitment may also have limited diversity of experience from within the sample."

Implications (pg. 18, lines 28-55)
This section lifts the study findings to become more general challenges related to improve the process of handover instructions from hospital to primary health care and increase the transferability of the results. And thereby supports the relevance of using behavioural science and the Theoretical Domains Framework as an analytical tool within the healthcare setting.
I will suggest that you refer to Michie et al 2014, which is already used as a reference.

This reference has been added as suggested.

Conclusion (Pg. 20 lines 1-16)
This conclusion does not add anything new to the aims/research questions of this study – only that you found ten influences across five TDF domains. I suggest that you add more information of the findings: e.g. which domains were found to be important, such as individual factors such as knowledge and skills, professional roles and identity, environmental and contextual factors and social and cultural aspects. (You could use parts of the implications section pg. 18 – lines 30-34).
I think that the conclusion should underline that improving the preparation of the handover instructions is a complex process that involve a lot of various processes and actors and is partly context sensitive and that potential future interventions must recognise this complexity.

The conclusion has been revised as below:

"This study identified ten likely important influences on preparations of discharge documentation by hospital staff for primary care teams, across five TDF domains. Important factors included: individual factors such as skills in writing discharge summaries, and knowledge of guidelines or what should be included; social factors including effective communication between professionals, learning from others and getting feedback; and environmental influences including noisy environments, software limitations and challenges, high workload, and staff changeovers leading to unfamiliarity with the patient. These influences represent potential targets for intervention designers to improve handovers between hospital and primary care and patient safety at discharge. This study demonstrated that preparation of handover documentation is a complex process, requiring multiple actors and different systems, and often takes place in busy, time-pressured environments. Interventions designed to improve the quality of handover documentation should recognise that complexity. Professional and public stakeholders should be involved in the intervention design to ensure it can work in real hospital settings and be effectively incorporated into clinical practice."

Response to Reviewer 2 comments:

I only have one major issue with it: the definition of a high-quality discharge document.
The first line of the abstract says "poor quality handover", and the conclusion refers to "quality of discharge documentation". If I search for quality, it appears 64 times in the document; it even appears on your definition of outcomes; however, you have not defined the quality of a discharge summary. This is problematic because your interview guide uses the term and, therefore, your participants defined "quality" intuitively without guarantee that everyone is talking about the same standard. For me, the quality of a discharge summary depends on its purpose. It should allow a smooth transition into primary care and avoid readmissions or emergency attendances. The professionals who are probably best placed to judge the adequacy or quality of a discharge summary are those who receive it and have to implement a treatment plan based on the information in the summary.

The following text has been added at the end of the first paragraph to add explanation about what is mean by poor quality (as you say, these relate to the purpose of the discharge summary, and were reported by those who receive them - GPs):

"The components of poor quality handover instructions were reported to be: missing key information; poor quality of written content, e.g. conflicting or incorrect information, instructions still in draft format; or unclear information about medications, therapeutic adjuncts or care equipment required, or follow-up arrangements and referrals."

We have added more information about how the interview was introduced to participants, which includes reference to the quality of information received by primary care teams:

"The interview started with an introduction to reasons for the interview, namely that the research team were interested in exploring more about how handover instructions are prepared ahead of a patient's discharge, and what factors may influence the quality of the handover instructions received by primary care and community teams."

The following text has been added to the limitations section:

"One limitation of this study is that the phrase "quality of handover instructions" was used in the topic guide, however the term "quality" was not clearly defined for participants. Therefore

they may have defined the term intuitively and may not all have been considering the same standard."

That said, the purpose of a discharge summary will be different for a PT or OT, and, to the best of my knowledge, there are no standard definitions of an adverse event due to poor quality handover for those professions. Maybe a fall within a week after discharge?
This also links to interviewing different professionals. This adds value, but including only one PT and one OT is problematic. Strictly speaking, these professionals report on different processes, and I find it more difficult to see the value of interviewing a single professional.

We are unaware of standard definitions of an adverse event due to poor quality handover instructions from a PT or OT.

The following text has been added to the limitations:

"While including different HPCs involved in preparing different forms of discharge documentation is a strength, the small numbers from different professional groups (and only having one physiotherapist and occupational therapist) is a limitation. However, there were many similarities in influences reported amongst participants across different professions. Future work could consider exploring differences in influences found between different professional groups."

There are also a couple of typos that I spotted:
Page 5, lines 25-26: "others were by introduced". Please, delete by
In Table 2, there is a quote from "junior doctor 5, interview 12" and another from "junior doctor 12, interview 12". I think there is a typo for one of them.

Thank you for finding these errors - they have been corrected.

Also, I could not find a statement about ethical approval or funding.

The following text has been added in the methods section:

"The research received ethical approval from the Health Research Authority, and Health and Care Research Wales (IRAS ID 242491). Following the written invitation to participate, and receipt of a participant information sheet, those who wished to take part gave written informed consent."

And a funding statement has been added at the end of the paper:

"This article is independent research funded by the National Institute for Health Research (NIHR) Imperial Biomedical Research Centre (grant number: 121520013) and the NIHR Imperial Patient Safety Translational Research Centre (grant number: PSTRC_2016_004). The views expressed in this publication are those of the authors and not necessarily those of the NHS, NIHR, or Department of Health and Social Care."

VERSION 2 - REVIEW

Reviewer 1

Name **Pedersen, Mona**

Affiliation **Aalborg University Hospital, Centre for Clinical Research, North Denmark Regional Hospital**

Date **29-Mar-2025**

COI

Thank you for the possibility to make a further review on the revised manuscript.

I find that the manuscript is revised thoroughly which has qualified the reporting and improved the readability of the manuscript.

Thus, I have only a few comments:

Introduction (pg. 3-4)

Pg 3 Line 21 – It is not necessary to use the abbreviation (GPs) to explain what is meant by primary care team – it is explained in line 11 (including GPs and pharmacists)

Pg 3 Line 48: You do not abbreviate physiotherapists and occupational therapists here, but the abbreviations are used later – pg. 5 line 3. Please consider whether it is necessary to use these abbreviations at all.

Pg. 4 line 14: You have previously (pg. 3 line 20) abbreviated Healthcare professionals (HCPs)

Methods (pg. 4-8)

Pg. 7 lines 5-8 (Outcomes)

You write that the influences likely to be most impactful are highlighted, together with their responding TDF domains – please clarify where and why?

Results (pg. 9-15)

Table 2 pg 12-13 – In the text (pg. 9 line 40-41) you write that you report on the ten most important influences – for transparency, I will suggest at you in the legend of Table 2 write that this table refers to the ten most important influences.

Limitations (pg. 17)

Pg 17, line 39 You write that involving both inductive and deductive elements is recommended in thematic analysis – however the reference by Atkins primarily addresses implementation research. Thus, I will suggest that you add "recommended in implementation research".

Reviewer **2**

Name **Castro-Avila, Ana**

Affiliation **University of York, Health Sciences**

Date **07-Apr-2025**

COI

Thanks for the opportunity to review this manuscript, which explores influences on the preparation of discharge documentation by different healthcare professionals.

I only have very minor revisions to suggest:

Page 11, lines 17-18: "They hadn’t previously met or didn’t know well" --> Contractions are usually not used in journal articles.

Page 17, lines 52-53: "would have further strengthened the validity of the conclusions" --> In qualitative research, we use "credibility" as the equivalent of "validity" in quantitative research.

Besides that, I am happy with the changes the authors made. Well done!

VERSION 2 - AUTHOR RESPONSE

Reviewer: 1

Reviewer comment	Response
Pg 3 Line 21 – It is not necessary to use the abbreviation (GPs) to explain what is meant by primary care team – it is explained in line 11 (including GPs and pharmacists)	This has been removed as advised.
Pg 3 Line 48: You do not abbreviate physiotherapists and occupational therapists here, but the abbreviations are used later – pg. 5 line 3. Please consider whether it is necessary to use these abbreviations at all.	These abbreviations have been removed.
Pg. 4 line 14: You have previously (pg. 3 line 20) abbreviated Healthcare professionals (HCPs)	This has been corrected.
Pg. 7 lines 5-8 (Outcomes)<br>You write that the influences likely to be most impactful are highlighted, together with their responding TDF domains – please clarify where and why?	The text has been edited as follows:<br>The influences likely to be most impactful are described in the text of the results as well as in Table 2, categorised according to their corresponding TDF domains. Categorisation by TDF domain can facilitate interpretation of findings, and support subsequent intervention design.
Results (pg. 9-15)<br>Table 2 pg 12-13 – In the text (pg. 9 line 40-41) you write that you report on the ten most important influences – for transparency, I will	This has been added to the legend.

suggest at you in the legend of Table 2 write that this table refers to the ten most important influences.	
Limitations (pg. 17)<br>Pg 17, line 39 You write that involving both inductive and deductive elements is recommended in thematic analysis – however the reference by Atkins primarily addresses implementation research. Thus, I will suggest that you add "recommended in implementation research".	This suggestion has been added.

Reviewer: 2

**Reviewer comment**	**Response**
Page 11, lines 17-18: "They hadn't previously met or didn't know well" --> Contractions are usually not used in journal articles.	This has been corrected.
Page 17, lines 52-53: "would have further strengthened the validity of the conclusions" --> In qualitative research, we use "credibility" as the equivalent of "validity" in quantitative research.	This term has been changed as suggested.
